# Supplementary material for: Quantifying within-city inequalities in child mortality across neighbourhoods in Accra, Ghana: a Bayesian spatial analysis
Source: BMJ Open. 2022 Jan 13;12(1):e054030. doi: 10.1136/bmjopen-2021-054030 (PMC8762100; doi:10.1136/bmjopen-2021-054030)
Supplement: Supplementary data [file bmjopen-2021-054030supp001.pdf]

Supplementary appendix 1 Relationship between number of children reported dead by women in a neighbourhood (as a proportion of live births) and the average number live births per woman, by age group of women. Each point represents a neighbourhood, coloured by district.

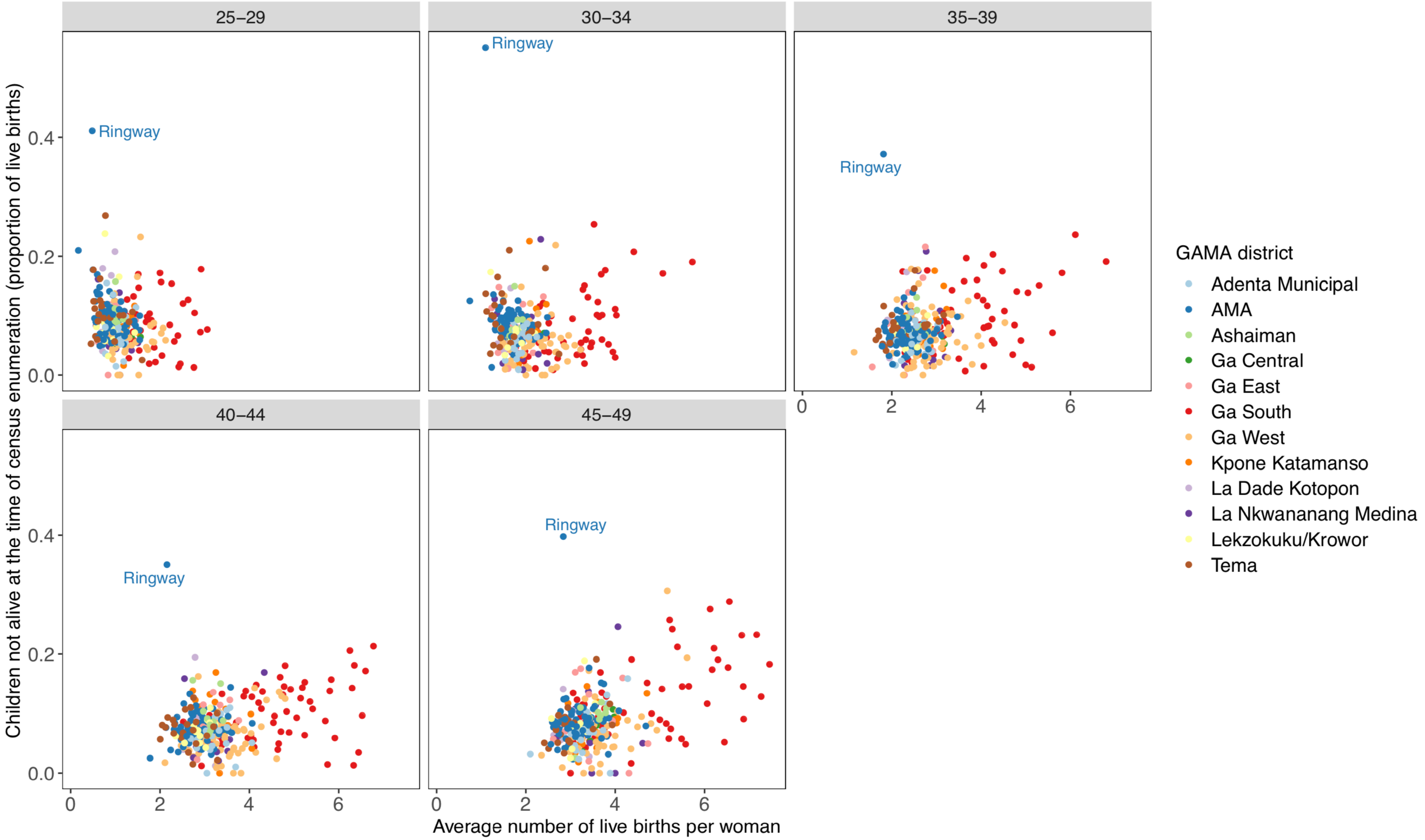

For each age group of women, only neighbourhoods in which women collectively reported more than 50 live births are shown. The Ringway neighbourhood was a consistent outlier across age groups of women and therefore we did not include the summary birth history data of women living in Ringway in our analysis. Reported estimates for Ringway were therefore informed only by data from surrounding neighbourhoods.
