# Supplementary material for: Quantifying within-city inequalities in child mortality across neighbourhoods in Accra, Ghana: a Bayesian spatial analysis
Source: BMJ Open. 2022 Jan 13;12(1):e054030. doi: 10.1136/bmjopen-2021-054030 (PMC8762100; doi:10.1136/bmjopen-2021-054030)
Supplement: Supplementary data [file bmjopen-2021-054030supp002.pdf]

Supplementary appendix 2. ‘Improved’ or ‘unimproved classification of census responses to questions on household characteristics.

**Household characteristic**	**Census response**	
	**‘improved’**	**‘unimproved’**
Dwelling type	Compound house (rooms)<br>Flat/Apartment<br>Semi-detached house<br>Separate house	Huts/Buildings (different compound)<br>Huts/Buildings (same compound)<br>Improvised home (kiosk/container, etc.)<br>Living quarters attached to office/shop<br>Tent<br>Uncompleted building<br>Other
Drinking water source	Bore-hole/Pump/Tube well<br>Bottled water<br>Pipe-borne inside dwelling<br>Pipe-borne outside dwelling<br>Protected spring<br>Protected well<br>Public tap/Standpipe<br>Sachet water	Dugout/Pond/Lake/Dam/Canal<br>Rainwater<br>River/Stream<br>Tanker supply/Vendor provided<br>Unprotected spring<br>Unprotected well<br>Other
Water source	Bore-hole/Pump/Tube well<br>Pipe-borne inside dwelling<br>Pipe-borne outside dwelling<br>Protected spring<br>Protected well<br>Public tap/Standpipe	Dugout/Pond/Lake/Dam/Canal<br>Rainwater<br>River/Stream<br>Tanker supply/Vendor provided<br>Unprotected spring<br>Unprotected well<br>Other
Toilet facilities	KVIP<br>Pit latrine<br>W.C.	Bucket/Pan<br>Public toilet (WC, KVIP, Pit, Pan, etc.)<br>No facilities (bush/beach/field)<br>Other
Solid waste disposal	Collected<br>Public dump (container)<br>Public dump (open space)	Buried by household<br>Burned by household<br>Dumped indiscriminately<br>Other
Liquid waste disposal	Through drainage into a pit (soak away)<br>Through drainage system into a gutter<br>Through the sewerage system	Thrown into gutter<br>Thrown onto compound<br>Thrown onto the street/outside<br>Other
Cooking fuel use	Electricity<br>Gas<br>Kerosene	Animal waste<br>Charcoal<br>Crop residue<br>Saw dust<br>Wood<br>None, no cooking<br>Other
Lighting source	Electricity (mains)<br>Electricity (private generator)<br>Solar energy	Candle<br>Crop residue<br>Firewood<br>Flashlight/Torch<br>Gas lamp<br>Kerosene lamp<br>Other
Floor material	Burnt brick<br>Cement/Concrete<br>Ceramic/Porcelain/Granite/Marble tiles<br>Stone<br>Terrazzo/Terrazzo tiles<br>Vinyl tiles	Earth/Mud<br>Wood<br>Other
Roof material	Cement/Concrete<br>Metal sheet<br>Roofing tile<br>Slate/Asbestos	Bamboo<br>Mud/Mud bricks/Earth<br>Thatch/Palm leaf or Raffia<br>Wood<br>Other
Wall material	Burnt bricks<br>Cement blocks/Concrete<br>Landcrete<br>Metal sheet/Slate/Asbestos<br>Stone	Bamboo<br>Mud brick/Earth<br>Palm leaf/Thatch (grass)/Raffia<br>Wood<br>Other
