# Supplementary material for: Quantifying within-city inequalities in child mortality across neighbourhoods in Accra, Ghana: a Bayesian spatial analysis
Source: BMJ Open. 2022 Jan 13;12(1):e054030. doi: 10.1136/bmjopen-2021-054030 (PMC8762100; doi:10.1136/bmjopen-2021-054030)

Supplementary appendix 3. Under-five mortality rates in neighbourhoods of the Greater Accra Metropolitan Area from sensitivity analyses replacing zero MAC-5q0 estimates with **a)** 0.0001 and **b)** the minimum estimated non-zero 5q0 (0.00632). Neighbourhood boundaries are shown in grey, district boundaries are shown in black. The colour scale diverges at the mean under-five mortality rate across all GAMA neighbourhoods. Neighbourhoods with U5M above the GAMA average are shown in orange; neighbourhoods with U5M below the GAMA average are shown in purple.

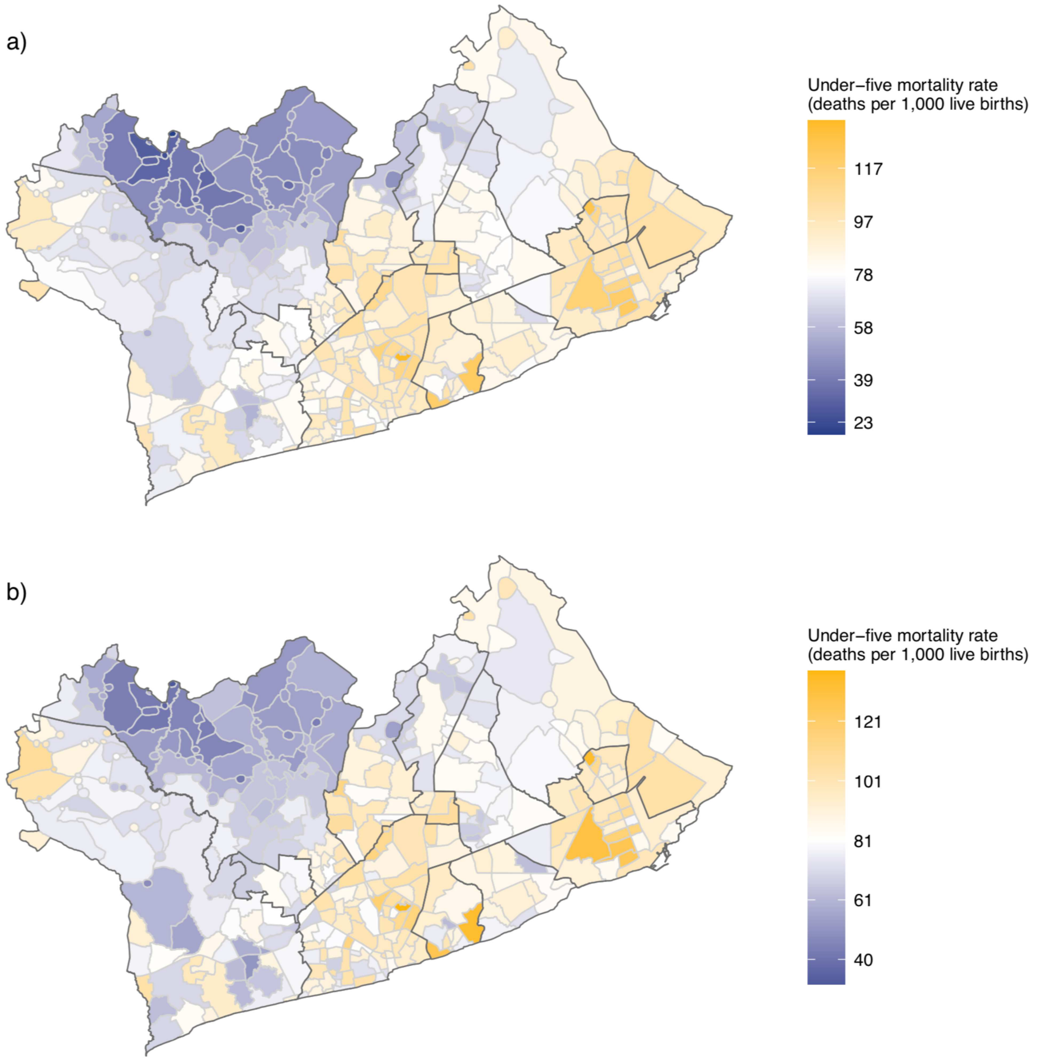

Supplement: Supplementary data [file bmjopen-2021-054030supp003.pdf]
