# Supplementary material for: Quantifying within-city inequalities in child mortality across neighbourhoods in Accra, Ghana: a Bayesian spatial analysis
Source: BMJ Open. 2022 Jan 13;12(1):e054030. doi: 10.1136/bmjopen-2021-054030 (PMC8762100; doi:10.1136/bmjopen-2021-054030)
Supplement: Supplementary data [file bmjopen-2021-054030supp004.pdf]

BMJ Publishing Group Limited (BMJ) disclaims all liability and responsibility arising from any reliance
 placed on this supplemental material which has been supplied by the author(s)

Supplementary appendix 4: The distribution of under−five mortality at increasing levels of neighbourhood living and socio−economic condition indicators.

Neighbourhoods are grouped into quintiles based on the measured indicator, separately by urban or peri−urban area. The horizontal line and box show the median and interquartile range.

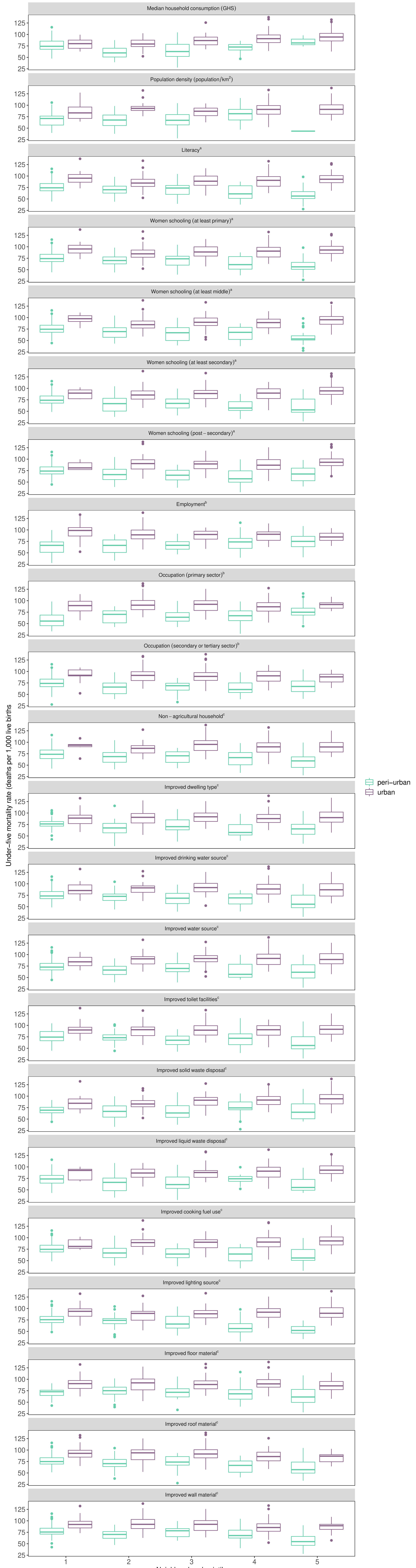

Neighbourhood indicator measured as
(a) the proportion of women of childbearing age;
(b) the proportion of population of working age (15–64 years); and,
(c) the proportion of total population.
