# Supplementary material for: Quantifying within-city inequalities in child mortality across neighbourhoods in Accra, Ghana: a Bayesian spatial analysis
Source: BMJ Open. 2022 Jan 13;12(1):e054030. doi: 10.1136/bmjopen-2021-054030 (PMC8762100; doi:10.1136/bmjopen-2021-054030)
Supplement: Author's manuscript [file bmjopen-2021-054030.draft_revisions.pdf]

BMJ Open is committed to open peer review. As part of this commitment we make the peer review history of every article we publish publicly available.

When an article is published we post the peer reviewers' comments and the authors' responses online. We also post the versions of the paper that were used during peer review. These are the versions that the peer review comments apply to.

The versions of the paper that follow are the versions that were submitted during the peer review process. They are not the versions of record or the final published versions. They should not be cited or distributed as the published version of this manuscript.

BMJ Open is an open access journal and the full, final, typeset and author-corrected version of record of the manuscript is available on our site with no access controls, subscription charges or pay-per-view fees (http://bmjopen.bmj.com).

If you have any questions on BMJ Open's open peer review process please email info.bmjopen@bmj.com

BMJ Open

Quantifying within-city inequalities in child mortality across neighbourhoods in Accra, Ghana.

Journal:	*BMJ Open*
Manuscript ID	bmjopen-2021-054030
Article Type:	Original research
Date Submitted by the Author:	01-Jun-2021

Complete List of Authors:	Bixby, Honor; McGill University,<br>Bennett, James; Imperial College London<br>Bawah, Ayaga; University of Ghana, Regional Institute for Population Studies<br>Arku, Raphael; University of Massachusetts Amherst<br>Annim, Samuel ; Ghana Statistical Service; University of Cape Coast<br>Anum, Jacqueline; Ghana Statistical Service<br>Mintah, Samilia; Ghana Statistical Service<br>Schmidt, Alexandra; McGill University<br>Agyei-Asabere, Charles; University of Ghana<br>Robinson, Brian; McGill University<br>Cavanaugh, Alicia; McGill University<br>Agyei-Mensah, Samuel; University of Ghana<br>Owusu, George; University of Ghana<br>Ezzati, Majid; Imperial College London; University of Ghana<br>Baumgartner, Jill ; McGill University

Keywords:	Community child health < PAEDIATRICS, EPIDEMIOLOGY, PUBLIC HEALTH

SCHOLARONE™
Manuscripts

I, the Submitting Author has the right to grant and does grant on behalf of all authors of the Work (as defined in the below author licence), an exclusive licence and/or a non-exclusive licence for contributions from authors who are: i) UK Crown employees; ii) where BMJ has agreed a CC-BY licence shall apply, and/or iii) in accordance with the terms applicable for US Federal Government officers or employees acting as part of their official duties; on a worldwide, perpetual, irrevocable, royalty-free basis to BMJ Publishing Group Ltd ("BMJ") its licensees and where the relevant Journal is co-owned by BMJ to the co-owners of the Journal, to publish the Work in this journal and any other BMJ products and to exploit all rights, as set out in our licence.

The Submitting Author accepts and understands that any supply made under these terms is made by BMJ to the Submitting Author unless you are acting as an employee on behalf of your employer or a postgraduate student of an affiliated institution which is paying any applicable article publishing charge ("APC") for Open Access articles. Where the Submitting Author wishes to make the Work available on an Open Access basis (and intends to pay the relevant APC), the terms of reuse of such Open Access shall be governed by a Creative Commons licence – details of these licences and which Creative Commons licence will apply to this Work are set out in our licence referred to above.

Other than as permitted in any relevant BMJ Author's Self Archiving Policies, I confirm this Work has not been accepted for publication elsewhere, is not being considered for publication elsewhere and does not duplicate material already published. I confirm all authors consent to publication of this Work and authorise the granting of this licence.

For peer review only

Quantifying within-city inequalities in child mortality across neighbourhoods in Accra, Ghana.

Honor Bixby[1,2*], James E. Bennett[3,4], Ayaga A. Bawah[5], Raphael E. Arku[6], Samuel K. Annim[7,8], Jacqueline D. Anum[7], Samilia E. Mintah[7], Alexandra M. Schmidt[1], Charles Agyei-Asabere[5], Brian E. Robinson[9], Alicia Cavanaugh[9], Samuel Agyei-Mensah[10], George Owusu[11], Majid Ezzati[3,4,5], Jill Baumgartner[1,2]

[1]Department of Epidemiology, Biostatistics and Occupational Health, McGill University, Montreal, Quebec, Canada.

[2]Institute for Health and Social Policy, McGill University, Montreal, Canada.

[3]Department of Epidemiology and Biostatistics, School of Public Health, Imperial College London, London, UK.

[4]MRC Centre for Environment and Health, Imperial College London, London, UK.

[5]Regional Institute for Population Studies, University of Ghana, Accra, Ghana.

[6]Department of Environmental Health Sciences, University of Massachusetts Amherst, Amherst, Massachusetts, USA.

[7]Ghana Statistical Service, Accra, Ghana.

[8]Department of Applied Economics, University of Cape Coast, Cape Coast, Ghana.

[9]Department of Geography, McGill University, Montreal, Quebec, Canada.

[10]Department of Geography and Resource Development, University of Ghana, Accra, Ghana.

[11]Institute of Statistical, Social and Economic Research, University of Ghana, Accra, Ghana.

*Correspondence to honor.bixby@mcgill.ca

For peer review only

Abstract

Objective Countries in sub-Saharan Africa suffer the highest rates of child mortality worldwide. Urban areas tend to have lower mortality than rural areas, but these comparisons likely mask large within-city inequalities. We aimed to quantify variation in child mortality across neighbourhoods of Accra – Ghana's capital city.

Methods We accessed data on >700,000 women aged 25-49 years living in the Greater Accra Metropolitan Area (GAMA) using the most recent Ghana census (2010). We summarised counts of child births and deaths by five-year age group of women and neighbourhood (n=406) and applied indirect demographic methods to convert the summaries to yearly probabilities of death before age five years. We fitted a Bayesian spatio-temporal model to the neighbourhood under-five mortality probabilities to obtain estimates for 2010, and examined correlations with indicators of neighbourhood living and socio-economic conditions.

Results Under-five mortality varied almost five-fold across neighbourhoods in GAMA in 2010, ranging from 28 (95% credible interval (CrI): 8-63) to 138 (95% CrI: 111-167) deaths per 1,000 live births. Under-five mortality was highest in the urban core and industrial districts, with a neighbourhood average of 95 deaths per 1000 live births. Peri-urban neighbourhoods performed better, on average, but had greater within-district variation (up to 3.8-fold). Under-five mortality was negatively correlated with multiple indicators of improved living and socio-economic conditions among peri-urban neighbourhoods. Among urban neighbourhoods, correlations were either weaker, not found, or, in some cases, reversed, including with median household consumption and women's schooling.

Conclusion Reducing child mortality in high burden urban neighbourhoods, where a substantial portion of the urban population resides, should be prioritised as part of continued efforts to meet the Sustainable Development Goal national target of less than 25 deaths per 1,000 live births.

For peer review only

Strength and limitations of this study

- We accessed the full microdata of latest census in Ghana that contained birth history data from over 700,000 women aged 25-49 years living in the Greater Accra Metropolitan Area.
- Georeferenced census data allowed estimation of under-five mortality at the fine spatial scale of neighbourhood with full coverage of the Greater Accra Metropolitan area.
- We used a flexible Bayesian spatial-temporal model that allowed each neighbourhood's mortality estimate to be informed by its own data and that of surrounding neighbourhoods and incorporated weights for the number of births recorded.
- Under-five mortality was indirectly calculated from summary birth history data because complete birth and death registration data were unavailable. These data may be subject to recall errors.
- No data were available on cause of death.

For peer review only

Background

Recent decades have delivered marked reductions in child mortality across all world regions.[1] Despite improvements, almost eight percent of all deaths globally in 2019 were children under five years of age,[2] mostly due to preventable and treatable causes linked to infection and malnutrition.[3] The rate of under-five mortality (U5M) in sub-Saharan Africa far exceeds other regions, estimated at 76 deaths per 1,000 live births in 2019, and under-five deaths in the region account for a growing proportion of the global total.[1]

On average, children living in cities across low- and middle-income regions have a survival advantage over their rural counterparts, largely due to improved education, employment and healthcare opportunities in urban areas.[4-6] While still predominantly rural, sub-Saharan Africa has the world's fastest growing urban population and cities are expected to absorb over 75% of the region's population growth over the next three decades.[7] This offers many opportunities for continued improvement in child mortality, however, infrastructure and service provision remain major challenges for cities in the region, exacerbated by the rapid pace of urban population growth.

The health benefits of urban living are not experienced equally by all city residents due in part to inequalities in the socioeconomic and living conditions between households and neighbourhoods. The mortality gap between slum versus non-slum urban dwellers in sub-Saharan Africa, for example, can be as large as the gap between urban and rural populations.[6, 8-10] Increased knowledge regarding the spatial heterogeneity of child mortality within cities at finer scales is necessary to target interventions and programs towards vulnerable populations, accelerate the progress necessary to reach the United Nations' Sustainable Development Goal (SDG) target of less than 25 deaths per 1,000 live births for all countries by 2030 (Goal 3, Target 3.2),[11] and better understand the drivers of such inequalities.[12]

With unique access to the complete records of the most recent Ghana Population and Housing Census,[13] we estimated under-five mortality (U5M) at the neighbourhood level across the Greater Accra Metropolitan Area (GAMA), providing insight into the magnitude of small area geographical inequalities in child mortality within a rapidly growing low-middle income city. This study was conducted within the Pathways to Equitable Healthy Cities study (http://equitablehealthycities.org/).

Methods

Study setting

For peer review only

Ghana is among the most urbanised countries in sub-Saharan Africa with an estimated urban population of over 18 million (58% of the total population) that is growing by ~3% each year.[7] GAMA is Ghana's administrative and economic capital and accounted for 29% of the country's urban population in 2010.[13] It covers ~1500 km^2 on the southern coast of the Greater Accra region. GAMA comprises 5019 census enumeration areas (EAs) – the smallest administrative geographical unit in Ghana – nested within 406 neighbourhoods, which were the geographical unit of analysis in this study, and 12 districts or 'municipalities' (**Figure 1**). The centrally located Accra Metropolitan Area (AMA) together with the more heavily industrialised Tema and Ashaiman municipalities in the east of the city contain the most densely populated neighbourhoods. A small fraction (~5%) of GAMA's population lives in areas classified as rural, mostly in northern GAMA and predominantly in the Ga West and Ga South districts.

Data

We accessed the full microdata of the most recent Ghana Population and Housing Census, conducted in 2010, via the Ghana Statistical Service. The census collects information on the number of children born and surviving at the time of survey, known as summary birth history, for all females aged 12 years and older. Other individual and household characteristics obtained in the census include employment status, occupational industry, schooling level, literacy, household amenities (including type of cooking fuel, drinking and non-drinking water source, sanitation facilities and waste disposal in use), dwelling type and structural features (including roof, floor, and wall materials), and EA of residence. Together, these provide information on the socioeconomic and living environments.

The EAs were georeferenced and assigned to neighbourhoods by the Ghana Statistical Service. We classified GAMA neighbourhoods as urban or peri-urban according to the census-derived designation of their constituent EAs as urban or rural, respectively. Most neighbourhoods (98%) comprised exclusively urban or rural EAs. We classified neighbourhoods that contained both urban and rural EAs as urban if over 50% of the population lived in urban EAs, and peri-urban otherwise (Figure 1).

To assess U5M, we summarised the birth history data of women of reproductive age (15-49 years) by five-year age group and neighbourhood. The Maternal Age Cohort (MAC) method was used to estimate the neighbourhood probability of death for children before the age of five (5q0) based on women's age, number of children ever born, proportion of children who have died and average parity.[14] This method outperforms alternatives for estimating U5M from

For peer review only

summary birth history data for subnational populations.[15] The method assigns a reference year to each 5q0 using maternal age as a proxy for duration of exposure to risk of death for a child. Children of young mothers are known to be at increased risk of death compared to the population average and younger women will have had fewer children, on average, than older women.[15] To avoid upward bias of our results and reduce instability of estimates due to low numbers of births, we therefore excluded 5q0 estimates derived from women aged 15-24 years. This left five 5q0 estimates for each neighbourhood (one for each five-year age group of women aged 25-49 years) covering a period from 1990 to 2005.

Statistical analysis

To obtain neighbourhood estimates of 5q0 for 2010, the year of the census, we fitted a Bayesian space-time model to the MAC-derived 5q0 estimates across all 2,030 neighbourhood-reference year units, transformed to the probit scale. The model included a linear time trend that could vary by neighbourhood. The time trend allowed data from different reference years, each of which is associated with a different age group, to inform the 5q0 in 2010. The neighbourhood intercepts and slopes were modelled using the Besag, York, and Mollié model,[16] where information is shared locally (i.e., amongst adjacent neighbourhoods) through spatially structured random effects with a conditional autoregressive prior, and globally through spatially unstructured Gaussian random effects. Neighbourhood-specific intercept and slope values were estimated by the sum of their respective spatially structured and spatially unstructured random effects. The prior distributions in the Bayesian framework allow the neighbourhood-specific parameters to be estimated by a neighbourhood's own data and data of contiguous neighbourhoods. This approach balances between overly unstable within-neighbourhood estimates and overly simplified aggregate estimates for all of GAMA. The reported estimate for the wealthy Ringway neighbourhood in AMA is informed entirely by data in bordering neighbourhoods as the data on child deaths were considered implausible (see **supplementary appendix 1**). In order to account for excess variability resulting from small numbers of children born to women in a given age-group and neighbourhood, we included a weighted variance term that gave more weight to estimates derived from a higher number of births. Samples from the posterior distributions of the intercepts and slopes were used to estimate 5q0 for the year 2010.

To avoid infinite values on the probit scale, we adjusted all MAC-derived 5q0 estimates of zero (n=146; 7%) to half the minimum estimated non-zero value across all units (0.00316). We

For peer review only

conducted sensitivity analyses to ensure our results were robust to this choice by replacing zero estimates with a lower value of 0.0001 and with the minimum estimated non-zero 5q0.

We monitored convergence using trace plots and obtained 5000 post burn-in samples from the posterior distributions of model parameters. We summarised the distributions of neighbourhood-specific parameters to report neighbourhood U5M estimates for 2010 with 95% credible intervals (CrI), representing the mean and the 2.5th and 97.5th percentiles of posterior samples, respectively. We present neighbourhood U5M estimates as deaths per 1,000 live births.

We calculated summary statistics of the individual and household characteristics within each neighbourhood to provide context for our mortality results. Household consumption, considered to provide a better indicator of living standards than income in low- and middle-income settings,[17 18] was estimated from the household socioeconomic and living environment characteristics recorded in the census. For this we used coefficient estimates from regression analyses of data on household consumption and the same household characteristics measured in the 2012 Ghana Living Standards survey.[19] We calculated the median household consumption as a measure of neighbourhood socioeconomic level. We additionally calculated population density; the proportion of the women of reproductive age (15-49 years) who were literate, had schooling to at least primary, middle, secondary and postsecondary levels; the proportion of the working age (15-64 years) population in any employment and in primary, secondary and tertiary sector occupations; and the proportion of households with indicators of improved living conditions (including dwelling type, materials of flooring, roofing and walls, methods of solid and liquid disposal, type of toilet facility, type of cooking fuel and type of drinking and other water source). Details of our classification of the census responses into indicators of 'improved' versus 'unimproved' living conditions are provided in the **supplementary appendix 2**.

We measured the correlations between posterior mean neighbourhood U5M estimates and socioeconomic and living environment indicators across all of the GAMA, and separately for urban versus peri-urban neighbourhoods, using the non-parametric Spearman's rank method.

All analyses were implemented in the open-source statistical software R version 3.6.1. The Bayesian model was implemented using the NIMBLE package version 0.9.1.

Patient and Public Involvement

The study used secondary data only.

For peer review only

Results

There were 713,581 women aged 25-49 years living in GAMA in 2010, who reported a total of 1,425,008 children, 1,312,030 (92.1%) of whom were alive at the time of the census. The number of women in this age range per neighbourhood ranged from 15 to 16,061 (median = 564). The proportion of their children born who had died ranged from 0 to 20% across neighbourhoods.

In 2010, the mean neighbourhood U5M rate in GAMA was 80 deaths per 1,000 live births (95% CrI 76-84). This compares to an estimated 69 deaths per 1000 live births nationally in Ghana.[1] Across all of GAMA, neighbourhood U5M varied almost five-fold, ranging from 28 deaths per 1,000 live births (95% CrI 8-63) in the Fantsenkor neighbourhood located in the north of GAMA to 138 deaths per 1,000 live births (95% CrI 111-167) in the Mamobi neighbourhood in the city's urban core (**Figure 2a and Figure 3**).

We found substantial variation in U5M between and within GAMA's twelve districts. Mean neighbourhood U5M was highest at 101 deaths per 1,000 live births in the Tema (95% CrI 89-113) and Ashaiman (95% CrI 89-114) municipalities situated in the southeast and east of GAMA, respectively. Both of these municipalities comprised only urban neighbourhoods. Mean neighbourhood U5M was lowest in the northern Ga West district (60 deaths per 1,000 live births, 95% CrI 52-68), where over 70% of neighbourhoods were considered peri-urban (**Figure 2a**). Although Ga West had the lowest mean mortality among GAMA districts, it had the highest within-district inequality (measured as the relative difference between the neighbourhood with the highest versus lowest mortality), with a 3.8-fold difference between the worst and best performing neighbourhoods. Within-district inequality was lowest in the Ga Central and Kpone Katamanso municipalities (1.4-fold difference across neighbourhoods).

The ten percent of neighbourhoods with the lowest U5M were concentrated in just three neighbouring districts in western GAMA, namely Ga West, Ga East and Ga South. The ten percent of neighbourhoods with the highest U5M were more dispersed, present in eight of the twelve GAMA districts, though almost half of highest mortality neighbourhoods fell within the urban core of AMA (**Figure 2b**). Neighbourhood mortality was consistently high in AMA where 80% of neighbourhoods (n=64) had U5M rates higher than the mean across GAMA neighbourhoods. This was true also for the Tema and Ashaiman municipalities, where 95% and 100% of neighbourhoods, respectively, had higher than average under-five mortality (**Figure 3**). The spatial pattern of U5M reported were unchanged in our sensitivity analyses

For peer review only

(**supplementary appendix 3**). The mean absolute difference in neighbourhood U5M estimates was 2.24 deaths per 1000 live births in our analysis replacing zero estimates of 5q0 with 0.0001, and -0.73 in our analysis replacing zero estimates with the minimum estimated non-zero 5q0 value.

The correlations between neighbourhood U5M and indicators of improved living and socio-economic conditions in peri-urban areas were distinct from urban neighbourhoods located mostly in the urban core and industrial areas (**Table 1**). In peri-urban areas, U5M was inversely correlated with most of the indicators of improved neighbourhood conditions analysed. The strongest associations were with the proportions of neighbourhood residents living in houses built with improved wall and roof materials, followed by the share of women aged 15-49 with at least primary or middle education or who were literate (Spearman's rho: -0.41 to -0.50). By comparison, correlations were weaker or reversed in urban neighbourhoods. Similar inverse correlations, though smaller in magnitude (Spearman's rho: -0.14 to -0.23), were found with the share of working age population engaged in secondary or tertiary sector occupations and the proportion of residents living in housing with improved wall and roof materials. No relationship was found for several indicators, including levels of women's literacy and access to improved drinking water source (Spearman's rho: 0.12 and -0.02, respectively). For others, there was a positive correlation, most notably with indicators of socioeconomic status, e.g., share of women educated to middle school level or above and average household consumption (Spearman's rho: 0.14 to 0.35).

Discussion

Our high spatial-resolution analysis of child mortality in GAMA, one of the largest metropolitan areas in sub-Saharan Africa, revealed considerable inequality in U5M with the highest levels in neighbourhoods located in the city's urban core and in more heavily industrial areas. Notably, even GAMA's lowest mortality neighbourhood had a mortality rate equivalent to the Dominican Republic and Bhutan which ranked 129th and 130th , respectively, among countries and territories worldwide in the UN's most recent estimates.[1] At present, no country has an estimated average child mortality rate as high as GAMA's highest mortality neighbourhoods.

A general pattern of higher child mortality extended across inner city and more industrial neighbourhoods despite higher median household consumption and generally higher levels of post-primary education among women in these neighbourhoods relative to their peri-urban

For peer review only

counterparts. Moreover, child mortality tended to be higher in better-off neighbourhoods of urban GAMA. It is possible that the spatial concentration of people across a wide socio-economic spectrum in dense inner-city neighbourhoods may generate what has been termed 'negative health externalities',[20] whereby localised inequalities compromise the real or perceived capacity of low-resourced individuals to sustain good health. Households that are better off may be negatively impacted by environmental degradation that is not bounded by household or neighbourhood boundaries, e.g., poor community sanitation or air pollution from neighbours' solid waste burning or use of household solid fuel stoves.[21-23]

Child mortality can be seen in some cases to follow historical patterns of socio-economic segregation in central Accra. The wealthier Cantonments neighbourhood – home to European settlers in the colonial era – was among the pockets of lower-than-average U5M (63 deaths per 1,000 live births), contrasted with higher rates found in low-income or 'slum' neighbourhoods, including Mamobi, Nima, New Town (83-138 deaths per 1,000 live births). However, other low-income neighbourhoods had U5M rates below the GAMA average, e.g., Jamestown and Sabon Zongo. The health penalty of areas with concentrated poor living conditions that arise from rapid, unplanned population growth, compiled by underinvestment in public services and infrastructure, in sub-Saharan African cities is well established and supported by previous studies conducted in central Accra.[6 8 24-26] Many such areas in Accra are neighbourhoods that historically comprised mostly native Ga people and African migrants. The variation seen across poor areas suggests distinct characteristics of these neighbourhoods and vulnerabilities of the people living in them.[21 27-30] Lower U5M rates in some of these neighbourhoods may reflect the success of targeted interventions to reduce mortality in the poorest communities.[31]

We observed the lowest rates of U5M in peri-urban neighbourhoods, where residents can still benefit from the urban services that contribute to improved child survival in cities – most births in Greater Accra, for example, take place in a health facility in the presence of a skilled health professional[32] – but avoid the potential hazards of inner-city living. A healthy selection effect may also contribute to the observed pattern of U5M where recent development of peri-urban neighbourhoods has encouraged wealthier residents to move out of the inner-city in search of better standards of living. Most of the areas considered peri-urban are newly developed neighbourhoods mostly occupied by middle- and high-income individuals. Our finding of lower U5M in neighbourhoods with improved socio-economic and living conditions in the peri-urban area is consistent with evidence linking child mortality to the household and neighbourhood environment. However, these reported correlations are intended only to give

For peer review only

broader context to the observed spatial patterns of U5M across GAMA and should be interpreted with caution.

A notable strength of our study is the fine spatial resolution of our U5M estimates, enabled by access to the full microdata of the 2010 census, including birth histories of over 700,000 women. Previous knowledge of child mortality inequalities in the metropolitan area was limited to broad district aggregates[33 34] or smaller areas in the urban core,[28 35] thereby excluding almost half of GAMA's total population, including residents of the rapidly developing Ga districts and the industrial Tema area.[36] In the absence of complete birth and death registration, summary birth history data used in our study can produce robust estimates of U5M[37] that are comparable to estimates directly calculated from complete birth history data that require detailed questionnaires.[15 38] The ease of summary birth history collection allows for its inclusion in national censuses and, in turn, analysis of U5M at finer spatial scales than is achievable through sample surveys.[39] Our analysis also makes important methodological advancements from available neighbourhood-level U5M estimates in GAMA.[28 35] Firstly we used a validated method for the use of summary birth history data in U5M estimation.[14 15] Secondly, we applied established Bayesian methods widely used in small area estimation[16] that allow sharing of information across spatial units to make robust estimates for our study neighbourhoods.

Our results show a different spatial pattern of U5M across GAMA compared with previous estimates reported at the district level that indicated higher U5M in the largely peri-urban Ga West district and minimal variability across the rest of GAMA.[34] This inconsistency could be due to a scarcity of data from areas outside the urban core used in the previous analysis. Their results rely heavily on data from the Demographic and Health Surveys that, for example in 2008, sampled 41 women living in only four EAs in Ga West compared with 299 women living in 38 EAs in the AMA. Estimates for Ga West would therefore be informed by data with limited geographical coverage within the district and data from bordering rural areas, where child mortality is on average higher.[6] By comparison, our study included data on children's births and deaths from 41,270 women living in all 92 neighbourhoods of GA West.

Our study has several limitations to be considered for future studies. Changes in GAMA over the last decade may mean our results do not reflect current patterns of U5M. Data from more recent sample surveys, however, lack the coverage, scope and sample sizes of the census which enabled us to conduct our analysis at very high spatial resolution. The georeferenced data in the 2021 Ghana census, which is currently in progress, can be used to provide up-to-date

For peer review only

estimates of U5M within GAMA and examine changes since 2010. As is the case for all studies of child mortality using census or sample survey data, we were limited to data on neighbourhood of residence at the time of the census and could not account for re-location since the death of a child. Finally, we acknowledge that intervention efforts to prevent child mortality would benefit from more detailed information on the underlying causes of these deaths and age at which GAMA children are most vulnerable.[40 41] In our study, we were unable to separately estimate neonatal, infant, or cause-specific mortality rates with the available census data. Investment in national civil registration systems across sub-Saharan Africa, including in Ghana, could provide these data in real-time and enable governments to monitor changes and progress in lowering child mortality and reducing inequalities.[42 43]

Conclusions

Our city-scale results contribute to a small but growing number of studies showing that within-city variation child mortality in sub-Saharan Africa can be as large as the difference between urban and rural areas or between countries.[6 8 10] Global targets for child mortality, including SDG 3.2, and related monitoring efforts focus entirely on national mortality rates.[11 44] While important for benchmarking countries' overall performance, progress towards SDG 3.2 does not automatically benefit high mortality and vulnerable population sub-groups within countries.[34 45] Our findings motivate more localised and increasingly disaggregated information on child deaths in order to deliver effective interventions and continue progress toward meeting national and international targets.

Universal access to high quality healthcare services can mitigate mortality inequalities in settings where children are born into different circumstances.[40 46 47] In many LMICs the rise in facility births has not produced the expected improvements in child mortality, demonstrating the importance of continued access to quality care throughout the early years of life.[46 48] Ghana's 2007-2015 Child Health Policy is among several national programs believed to have contributed to reductions in overall child mortality and in inequalities between subregions by scaling up interventions with proven efficacy to prevent child deaths, including, for example, oral rehydration therapy and zinc for treatment of diarrhoea, vitamin A supplementation and antibiotic treatment for pneumonia.[49 50] As Ghana continues to urbanise, its cities and metropolitan areas, including GAMA, will play an increasingly important role in building on national child survival efforts. Our study identified neighbourhoods with high child mortality in GAMA's central and industrial areas, where the urban poor may still face financial and physical barriers to accessing health services.[51 52] While on average lower, child mortality in

For peer review only

peri-urban neighbourhoods was more variable and correlated with living and socio-economic conditions. Complementary investment in developing infrastructure and services in neighbourhoods outside of the urban core, while ensuring that conditions in densely populated central and industrial areas do not deteriorate, could further contribute to improving child mortality and promoting health equity.

For peer review only

Contributions

HB, ME and JB developed the study concept. HB, AAB, SKA, JDA, SEM, AC and SAM contributed to data collation. HB conducted the statistical analysis with input from JEB, REA, AS, BER, ME and JB. HB, AB, REA, SAM, BER, ME and JB contributed to data interpretation. HB wrote the first draft of the manuscript with input from JB. All authors contributed to revising and finalising the manuscript.

Ethics statement

The study used secondary data only.

Funding

This work is supported by the Pathways to Equitable Healthy Cities grant from the Wellcome Trust [209376/Z/17/Z]. HB is supported by a Canadian Institute of Health Research Banting Postdoctoral Fellowship.

Competing interests

None to declare.

Data availability

Estimates of neighbourhood U5M will be made available at http://equitablehealthycities.org/data-download/. The full microdata of the 2010 Ghana Population and Housing Census are with the Ghana Statistical Service and are not publicly available. Data from a random 10% sample of households enumerated in the 2010 Population and Housing census are publicly available and can be downloaded from Ghana Statistical Services online data catalogue (https://www2.statsghana.gov.gh/nada/index.php/catalog/51).

Code availability

The Bayesian model code will be made available at http://equitablehealthycities.org/data-download/.

For peer review only

References

1. United Nations Inter-agency Group for Child Mortality Estimation. Levels & Trends in Child Mortality. New York: UN, 2020.
2. Wang H, Abbas KM, Abbasifard M, et al. Global age-sex-specific fertility, mortality, healthy life expectancy (HALE), and population estimates in 204 countries and territories, 1950–2019: a comprehensive demographic analysis for the Global Burden of Disease Study 2019. *Lancet* 2020;396(10258):1160-203. doi: 10.1016/S0140-6736(20)30977-6
3. GBD 2015 Child Mortality Collaborators. Global, regional, national, and selected subnational levels of stillbirths, neonatal, infant, and under-5 mortality, 1980-2015: a systematic analysis for the Global Burden of Disease Study 2015. *The Lancet* 2016;388(10053):1725-74. doi: 10.1016/S0140-6736(16)31575-6
4. Balk D, Pullum T, Storeygard A, et al. A spatial analysis of childhood mortality in West Africa. *Popul Space Place* 2004;10(3):175-216. doi: 10.1002/psp.328
5. Corker J. Fertility and child mortality in urban West Africa: leveraging geo-referenced data to move beyond the urban/rural dichotomy. *Popul Space Place* 2017;23(3):e2009. doi: 10.1002/psp.2009 [published Online First: 2017/09/26]
6. Fink G, Günther I, Hill K. Slum residence and child health in developing countries. *Demography* 2014;51(4):1175-97. doi: 10.1007/s13524-014-0302-0 [published Online First: 2014/06/05]
7. United Nations Department of Economic and Social Affairs PD. World urbanization prospects: the 2018 revision. New York: UN, 2019.
8. Günther I, Harttgen K. Deadly cities? Spatial inequalities in mortality in sub-Saharan Africa. *Popul Dev Rev* 2012;38(3):469-86. doi: 10.1111/j.1728-4457.2012.00512.x
9. Kyu HH, Shannon HS, Georgiades K, et al. Association of urban slum residency with infant mortality and child stunting in low and middle income countries. *Biomed Res Int* 2013;2013:604974. doi: 10.1155/2013/604974
10. Timæus IM, Lush L. Intra-urban differentials in child health. *Health Transit Rev* 1995;5(2):163-90.
11. United Nations. Transforming our world: the 2030 agenda for sustainable development. New York: UN, 2015.
12. Bhutta ZA. Mapping the geography of child mortality: a key step in addressing disparities. *Lancet Glob Health* 2016;4(12):e877-e78. doi: 10.1016/S2214-109X(16)30264-9
13. Ghana Statistical Service (GSS). Ghana Population and Housing Census. In: Service GS, ed. Accra, Ghana, 2010.
14. Rajaratnam JK, Tran LN, Lopez AD, et al. Measuring under-five mortality: validation of new low-cost methods. *PLoS Med* 2010;7(4):e1000253. doi: 10.1371/journal.pmed.1000253
15. Verhulst A. Child mortality estimation: An assessment of summary birth history methods using microsimulation. *Demographic Research* 2016;34:1075-128. doi: 10.4054/DemRes.2016.34.39
16. Besag J, York J, Mollié A. Bayesian image restoration, with two applications in spatial statistics. *Ann Inst Stat Math* 1991;1:1-20. doi: 10.1007/BF00116466
17. Deaton A. The analysis of household surveys: a microeconometric approach to development policy. Baltimore, MD: Published for the World Bank [by] Johns Hopkins University Press, 1997.
18. Deaton A, Zaidi S. Guidelines for constructing consumption aggregates for welfare analysis. LSMS Working Paper No. 135. Washington, D.C.: World Bank 2002.

For peer review only

19. Cavanaugh A, Robinson BE, Bixby H, et al. Locating poverty and inequality: an application of small area estimation methods using survey and census data from Ghana. *Manuscript in preparation* 2021
20. Montgomery M, Hewett PC. Urban poverty and health in developing countries: household and neighborhood effects. *Demography* 2005;42(3):397–425. doi: 10.1353/dem.2005.0020
21. Owusu G. Social effects of poor sanitation and waste management on poor urban communities: a neighborhood-specific study of Sabon Zongo, Accra. *J Urban* 2010;3(2):145-60. doi: 10.1080/17549175.2010.502001
22. Guiteras R, Levinsohn J, Mobarak AM. Encouraging sanitation investment in the developing world: A cluster-randomized trial. *Science* 2015;348(6237):903-06. doi: 10.1126/science.aaa0491
23. Zhou Z, Dionisio KL, Verissimo TG, et al. Chemical composition and sources of particle pollution in affluent and poor neighborhoods of Accra, Ghana. *Environ Res Lett* 2013;8(4):044025. doi: 10.1088/1748-9326/8/4/044025
24. Jorgenson AK, Rice J. Urban slum growth and human health: a panel study of infant and child mortality in less-developed countries, 1990–2005. *J Poverty* 2010;14(4):382-402. doi: 10.1080/10875549.2010.517073
25. Rice J, Rice JS. The concentration of disadvantage and the rise of an urban penalty: urban slum prevalence and the social production of health inequalities in the developing countries. *Int J Health Serv* 2009;39(4):749-70. doi: 10.2190/HS.39.4.i [published Online First: 2009/11/26]
26. Ezeh A, Oyebode O, Satterthwaite D, et al. The history, geography, and sociology of slums and the health problems of people who live in slums. *Lancet* 2017;389(10068):547-58. doi: 10.1016/S0140-6736(16)31650-6
27. Jankowska MM, Weeks JR, Engstrom R. Do the most vulnerable people live in the worst slums? A spatial analysis of Accra, Ghana. *Ann GIS* 2011;17(4):221-35. doi: 10.1080/19475683.2011.625976
28. Jankowska MM, Benza M, Weeks JR. Estimating spatial inequalities of urban child mortality. *Demogr Res* 2013;28:33-62. doi: 10.4054/DemRes.2013.28.2
29. Hewett PC, Montgomery MR. Poverty and public services in developing-country cities. Policy Research Division Working Paper No. 154. New York: Population Council 2001.
30. Temin M, Montgomery M, Engebretsen S, et al. Girls on the move: adolescent girls & migration in the developing world. New York: Population Council, 2013.
31. UNICEF, Institute of Statistical Social and Economic Research (ISSER) University of Ghana, Carolina Population Center University of North Carolina at Chapel Hill, et al. Ghana LEAP 1000 Programme: Endline Evaluation Report. Ghana: Ministry of Gender, Children and Social Protection, 2018.
32. Ghana Statistical Service (GSS), Ghana Health Service (GHS), ICF International. Ghana Demographic and Health Survey 2014. Rockville, Maryland, USA: GSS, GHS, and ICF International, 2015.
33. Arku RE, Bennett JE, Castro MC, et al. Geographical inequalities and social and environmental risk factors for under-five mortality in Ghana in 2000 and 2010: Bayesian spatial analysis of census data. *PLoS Med* 2016;13(6):e1002038. doi: 10.1371/journal.pmed.1002038
34. Burstein R, Henry NJ, Collison ML, et al. Mapping 123 million neonatal, infant and child deaths between 2000 and 2017. *Nature* 2019;574(7778):353-58. doi: 10.1038/s41586-019-1545-0

For peer review only

35. Toure S, Weeks J, Lopez-Carr D, et al. Evaluating links between dynamic urban landscapes and under-five child mortality in Accra, Ghana. *Demogr Res* 2020;42(20):589-614. doi: 10.4054/DemRes.2020.42.20
36. Grant R, Yankson P. Accra. *Cities* 2003;20(1):65-74. doi: 10.1016/s0264-2751(02)00090-2
37. Hill K, Lopez AD, Shibuya K, et al. Interim measures for meeting needs for health sector data: births, deaths, and causes of death. *Lancet* 2007;370(9600):1726-35. doi: 10.1016/S0140-6736(07)61309-9
38. Silva R. Child mortality estimation: consistency of under-five mortality rate estimates using full birth histories and summary birth histories. *PLoS Med* 2012;9(8):e1001296. doi: 10.1371/journal.pmed.1001296
39. Li Z, Hsiao Y, Godwin J, et al. Changes in the spatial distribution of the under-five mortality rate: small-area analysis of 122 DHS surveys in 262 subregions of 35 countries in Africa. *PLoS One* 2019;14(1):e0210645. doi: 10.1371/journal.pone.0210645
40. Bhutta ZA, Das JK, Bahl R, et al. Can available interventions end preventable deaths in mothers, newborn babies, and stillbirths, and at what cost? *Lancet* 2014;384(9940):347-70. doi: 10.1016/s0140-6736(14)60792-3
41. Keats EC, Das JK, Salam RA, et al. Effective interventions to address maternal and child malnutrition: an update of the evidence. *Lancet Child Adolesc Health* 2021 doi: 10.1016/s2352-4642(20)30274-1
42. Eilerts H, Romero Prieto J, Eaton JW, et al. Age patterns of under-5 mortality in sub-Saharan Africa during 1990–2018: A comparison of estimates from demographic surveillance with full birth histories and the historic record. *Demogr Res* 2021;44:415-42. doi: 10.4054/DemRes.2021.44.18
43. Lopez AD, McLaughlin D, Richards N. Reducing ignorance about who dies of what: research and innovation to strengthen CRVS systems. *BMC Med* 2020;18(1):58. doi: 10.1186/s12916-020-01526-9 [published Online First: 2020/03/10]
44. United Nations. The Millennium Development Goals report 2015. New York: UN, 2015.
45. Burke M, Heft-Neal S, Bendavid E. Sources of variation in under-5 mortality across sub-Saharan Africa: a spatial analysis. *Lancet Glob Health* 2016;4(12):e936-e45. doi: 10.1016/s2214-109x(16)30212-1 [published Online First: 2016/10/30]
46. Roder-DeWan S, Nimako K, Twum-Danso NAY, et al. Health system redesign for maternal and newborn survival: rethinking care models to close the global equity gap. *BMJ Glob Health* 2020;5(10):e002539. doi: 10.1136/bmjgh-2020-002539 [published Online First: 2020/10/16]
47. Kruk ME, Gage AD, Joseph NT, et al. Mortality due to low-quality health systems in the universal health coverage era: a systematic analysis of amenable deaths in 137 countries. *Lancet* 2018;392(10160):2203-12. doi: 10.1016/s0140-6736(18)31668-4
48. Campbell OMR, Calvert C, Testa A, et al. The scale, scope, coverage, and capability of childbirth care. *Lancet* 2016;388(10056):2193-208. doi: 10.1016/s0140-6736(16)31528-8
49. Ministry of Health G. Under five's child health policy: 2007-2015. Accra, Ghana: Ministry of Health, Ghana, 2007.
50. Kayode GA, Grobbee DE, Koduah A, et al. Temporal trends in childhood mortality in Ghana: impacts and challenges of health policies and programs. *Glob Health Action* 2016;9:31907-07. doi: 10.3402/gha.v9.31907
51. Blanchet NJ, Fink G, I O-A. The effect of Ghana's National Health Insurance Scheme on health care utilisation. *Ghana Med J* 2012;46(2):76-84.
52. Earth Institute Millennium Cities Initiative. AMA community upgrading profile: Nima-Maamobi drain area. New York: The Earth Institute at Columbia University, 2012.

For peer review only

For peer review only

Table 1 Population and household characteristics of neighbourhoods in the Greater Accra Metropolitan Area and correlations with neighbourhood under-five mortality in 2010. Median levels across all, peri-urban and urban neighbourhoods presented with interquartile range in parentheses. Spearman's rank correlation coefficients show the correlation between neighbourhood under-five mortality and neighbourhood characteristics that indicate the socio-economic and living conditions. Supplementary appendix 4 shows the distribution of under-five mortality across quintiles of analysed indicators.

Neighbourhood population and household characteristics	Median (inter-quartile range)			Correlation coefficient (Spearman's rho)		
	all	peri-urban	urban	all	peri-urban	urban
Child mortality rate (deaths per 1,000 live births)	80 (68-92)	69 (57-79)	90 (79-99)	n/a	n/a	n/a
Population	2972 (767-12798)	730 (439-1380)	11754 (6254-20873)	n/a	n/a	n/a
Population aged under five years	384 (107-1430)	105 (64-195)	1267 (703-2486)	n/a	n/a	n/a
Median household consumption (GHS)	5599 (4249-6894)	4485 (3616-5432)	6690 (5633-7473)	0.44*	-0.18*	0.35*
Population density (per km^2)	1858 (614-6366)	682 (156-1405)	5610 (1914-14513)	0.44*	0.04	0.09
Women's s literacy[a]	90.2% (83.2%-93.3%)	88.0% (68.9%-93.0%)	91.4% (88.2%-93.4%)	0.03	-0.41*	0.12
Women's schooling: at least primary[a]	90.2% (83.2%-93.3%)	88.0% (68.9%-93.0%)	91.4% (88.2%-93.4%)	0.03	-0.41*	0.12
Women's schooling: at least middle[a]	78.7% (67.9%-83.4%)	74.9% (43.3%-81.9%)	80.4% (76.0%-84.2%)	0.09	-0.41*	0.14*
Women's schooling: at least secondary[a]	36.6% (24.5%-44.3%)	30.7% (6.4%-39.8%)	40.6% (33.3%-47.7%)	0.18*	-0.38*	0.21*
Women's schooling: post-secondary[a]	10.5% (4.9%-15.4%)	7.4% (1.2%-12.5%)	12.6% (8.3%-17.6%)	0.18*	-0.30*	0.17*
Employment[b]	67.4% (64.7%-70.5%)	68.2% (64.5%-72.9%)	67.1% (64.9%-68.9%)	-0.01	0.31*	-0.18*
Occupation: primary sector[b]	2.0% (1.5%-3.8%)	3.4% (1.6%-21.4%)	1.8% (1.4%-2.5%)	-0.04	0.44*	-0.03
Occupation: non-primary sector[b]	64.0% (60.3%-66.6%)	61.7% (37.5%-65.9%)	64.9% (62.6%-66.7%)	0.04	-0.25*	-0.14*
Non-agricultural household[c]	94.4% (88.8%-96.4%)	89.1% (47.2%-95.3%)	95.3% (93.3%-96.9%)	0.10*	-0.40*	0.03
Improved dwelling type[c]	88.1% (79.4%-93.4%)	88.5% (78.1%-94.7%)	88.1% (80.0%-92.3%)	-0.13*	-0.28*	0.02
Improved drinking water source[c]	95.1% (85.4%-97.9%)	92.8% (78.6%-99.0%)	95.9% (89.7%-97.6%)	-0.11*	-0.39*	-0.02
Improved water source[c]	89.5% (71.8%-96.7%)	80.5% (50.6%-94.6%)	93.7% (81.7%-97.1%)	0.12*	-0.18*	0.07
Improved toilet facilities[c]	70.2% (48.3%-84.6%)	74.2% (51.2%-89.7%)	66.5% (47.3%-80.6%)	-0.18*	-0.29*	0.00

For peer review only

Neighbourhood population and household characteristics	Median (inter-quartile range) all	peri-urban	urban	Correlation coefficient (Spearman's rho) all	peri-urban	urban
Improved solid waste disposal[c]	77.4% (54.2%-90.8%)	64.4% (36.6%-86.3%)	85.7% (68.6%-93.4%)	0.34*	0.05	0.26*
Improved liquid waste disposal[c]	26.3% (10.2%-40.0%)	12.7% (1.9%-26.5%)	32.9% (23.8%-48.3%)	0.31*	-0.25*	0.27*
Improved cooking fuel use[c]	45.0% (27.4%-55.2%)	35.9% (4.8%-50.2%)	49.2% (39.4%-57.9%)	0.12*	-0.37*	0.13*
Improved lighting source[c]	83.9% (71.9%-92.0%)	79.8% (55.2%-87.6%)	88.4% (77.6%-93.3%)	0.05	-0.48*	0.1
Improved floor material[c]	90.9% (84.6%-94.1%)	92.1% (84.4%-96.2%)	90.3% (84.9%-93.0%)	-0.14*	-0.18*	-0.05
Improved roof material[c]	95.6% (91.2%-97.2%)	95.2% (79.8%-98.3%)	95.6% (93.6%-96.7%)	-0.25*	-0.43*	-0.23*
Improved wall material[c]	87.2% (76.0%-92.4%)	85.9% (59.2%-93.8%)	87.6% (83.0%-91.7%)	-0.22*	-0.50*	-0.18*

* significant at the 5% level.

[a] measured as the proportion of women of childbearing age (15-49 years) in the neighbourhood.

[b] measured as the proportion of population of working age (15-64 years) in the neighbourhood.

[c] measured as the proportion of population in the neighbourhood.

For peer review only

Figure 1. The Greater Accra Metropolitan Area with neighbourhood boundaries shown in grey and district boundaries shown in black (grey) (source: Ghana Statistical service). Urban neighbourhoods are shown in purple and peri-urban neighbourhoods shown in green. The inset shows the location of GAMA in Ghana and Western Africa.

For peer review only

Figure 2. a) Neighbourhood under-five mortality in the Greater Accra Metropolitan Area by district. The black vertical line and box show the median and interquartile range of under-five mortality across neighbourhoods in a district and the red vertical line shows the mean. b) The contribution of neighbourhoods in each district to each decile of neighbourhood child mortality. Decile 1 groups the 10% of neighbourhoods with the lowest child mortality rates and decile 10 groups the 10% of neighbourhoods with the highest child mortality rates.

For peer review only

Figure 3. Under-five mortality rates in neighbourhoods of the Greater Accra Metropolitan Area in 2010. Neighbourhood boundaries are shown in grey, district boundaries are shown in black. The colour scale diverges at the mean under-five mortality rate across all GAMA neighbourhoods. Neighbourhoods with U5M above the GAMA average are shown in orange; neighbourhoods with U5M below the GAMA average are shown in purple.

For peer review only

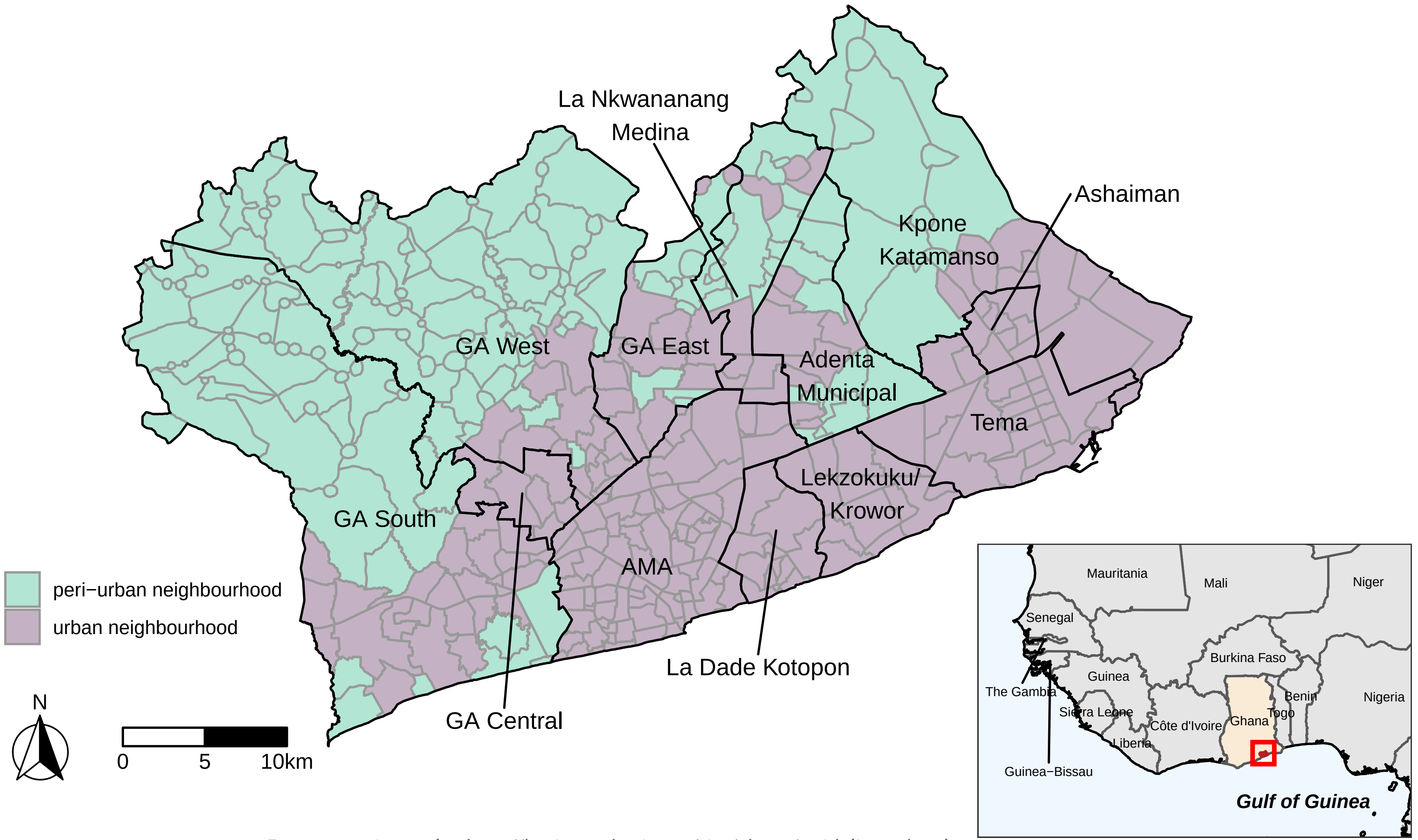
La Nkwananang
Medina
Ashaiman
Kpone
Katamanso
GA West
GA East
Adenta
Municipal
Tema
Lekzokuku/
Krowor
GA South
AMA
La Dade Kotopon
GA Central
peri−urban neighbourhood
urban neighbourhood
N
0
5
10km
Mauritania
Mali
Niger
Senegal
Burkina Faso
Guinea
The Gambia
Benin
Nigeria
Sierra Leone
Togo
Ghana
Côte d'Ivoire
Liberia
Guinea−Bissau
Gulf of Guinea

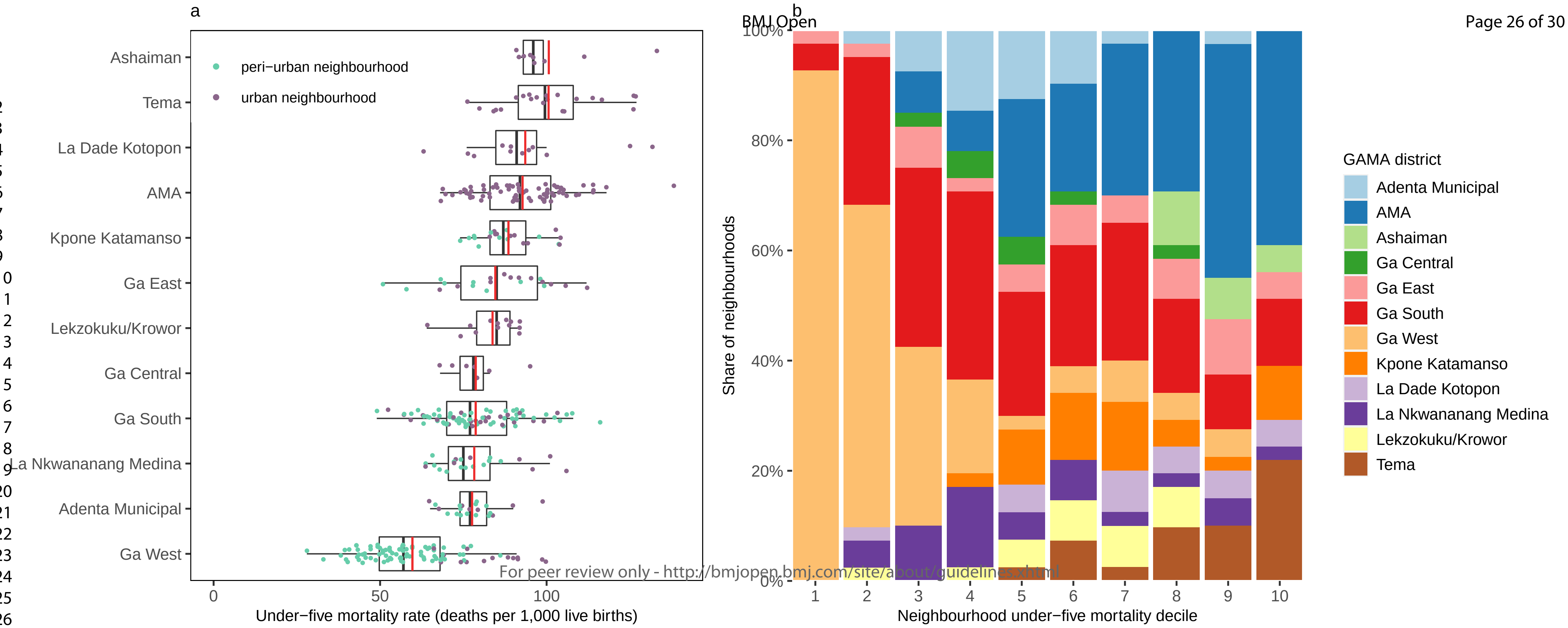
a
Ashaiman
Tema
La Dade Kotopon
AMA
Kpone Katamanso
Ga East
Lekzokuku/Krowor
Ga Central
Ga South
La Nkwananang Medina
Adenta Municipal
Ga West
peri−urban neighbourhood
urban neighbourhood
0
50
100
Under−five mortality rate (deaths per 1,000 live births)
b
100%
80%
60%
40%
20%
0%
Share of neighbourhoods
1
2
3
4
5
6
7
8
9
10
Neighbourhood under−five mortality decile
GAMA district
Adenta Municipal
AMA
Ashaiman
Ga Central
Ga East
Ga South
Ga West
Kpone Katamanso
La Dade Kotopon
La Nkwananang Medina
Lekzokuku/Krowor
Tema

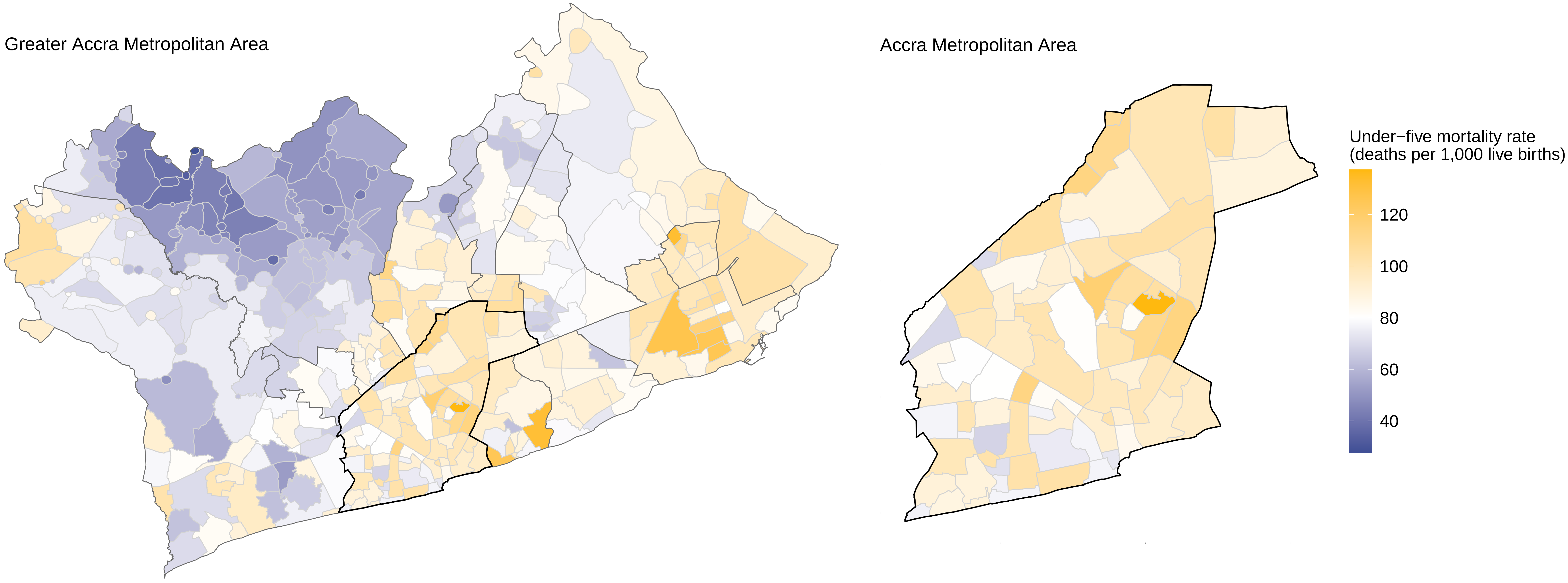
Greater Accra Metropolitan Area
Accra Metropolitan Area
Under−five mortality rate
(deaths per 1,000 live births)
120
100
80
60
40

Supplementary appendix 1 Relationship between number of children reported dead by women in a neighbourhood (as a proportion of live births) and the average number live births per woman, by age group of women. Each point represents a neighbourhood, coloured by district.

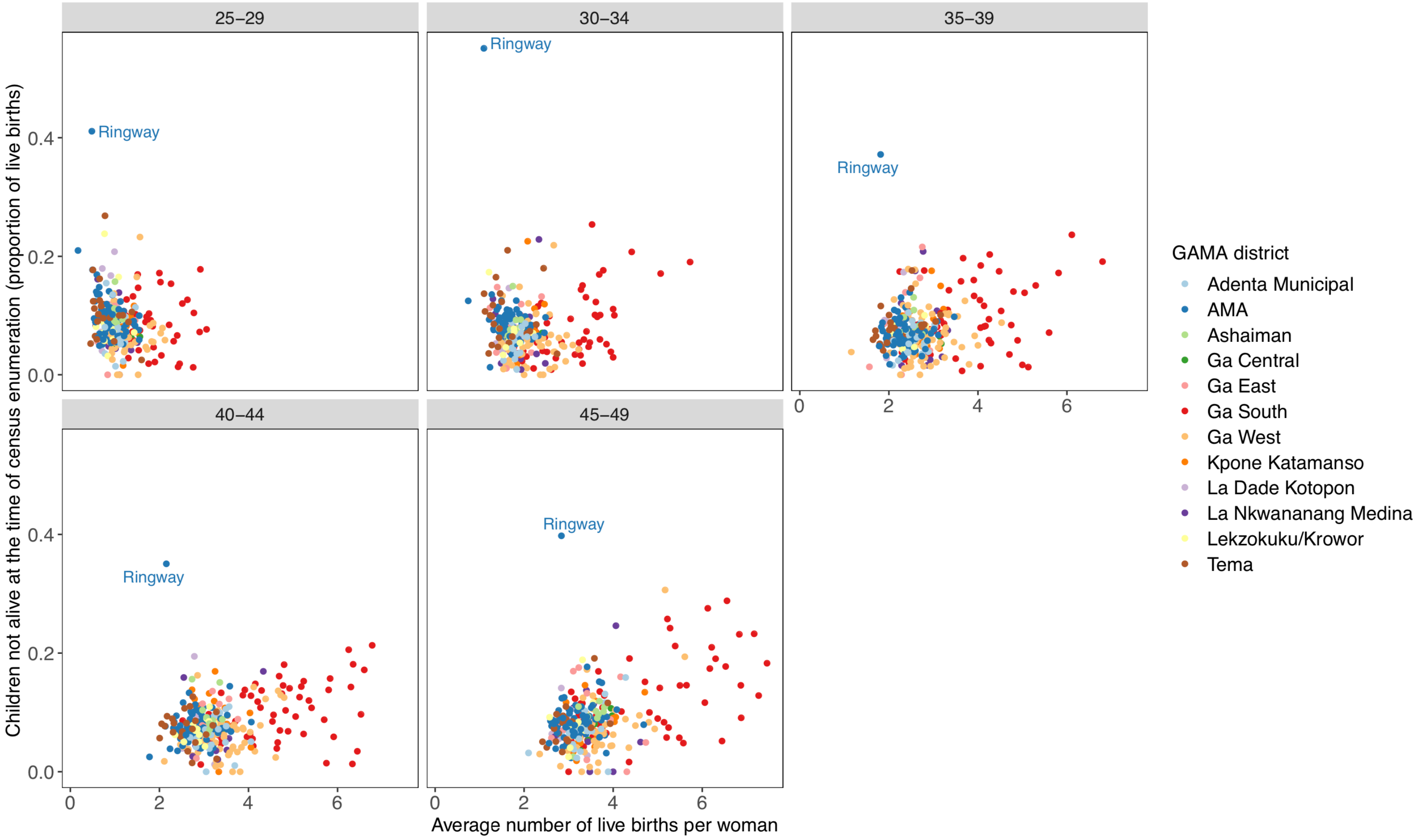

For each age group of women, only neighbourhoods in which women collectively reported more than 50 live births are shown. The Ringway neighbourhood was a consistent outlier across age groups of women and therefore we did not include the summary birth history data of women living in Ringway in our analysis. Reported estimates for Ringway were therefore informed only by data from surrounding neighbourhoods.

Supplementary appendix 2. 'Improved' or 'unimproved classification of census responses to questions on household characteristics.

Household characteristic	Census response	
	'improved'	'unimproved'
Dwelling type	Compound house (rooms)<br>Flat/Apartment<br>Semi-detached house<br>Separate house	Huts/Buildings (different compound)<br>Huts/Buildings (same compound)<br>Improvised home (kiosk/container, etc.)<br>Living quarters attached to office/shop<br>Tent<br>Uncompleted building<br>Other
Drinking water source	Bore-hole/Pump/Tube well<br>Bottled water<br>Pipe-borne inside dwelling<br>Pipe-borne outside dwelling<br>Protected spring<br>Protected well<br>Public tap/Standpipe<br>Sachet water	Dugout/Pond/Lake/Dam/Canal<br>Rainwater<br>River/Stream<br>Tanker supply/Vendor provided<br>Unprotected spring<br>Unprotected well<br>Other
Water source	Bore-hole/Pump/Tube well<br>Pipe-borne inside dwelling<br>Pipe-borne outside dwelling<br>Protected spring<br>Protected well<br>Public tap/Standpipe	Dugout/Pond/Lake/Dam/Canal<br>Rainwater<br>River/Stream<br>Tanker supply/Vendor provided<br>Unprotected spring<br>Unprotected well<br>Other
Toilet facilities	KVIP<br>Pit latrine<br>W.C.	Bucket/Pan<br>Public toilet (WC, KVIP, Pit, Pan, etc.)<br>No facilities (bush/beach/field)<br>Other
Solid waste disposal	Collected<br>Public dump (container)<br>Public dump (open space)	Buried by household<br>Burned by household<br>Dumped indiscriminately<br>Other
Liquid waste disposal	Through drainage into a pit (soak away)<br>Through drainage system into a gutter<br>Through the sewerage system	Thrown into gutter<br>Thrown onto compound<br>Thrown onto the street/outside<br>Other
Cooking fuel use	Electricity<br>Gas<br>Kerosene	Animal waste<br>Charcoal<br>Crop residue<br>Saw dust<br>Wood<br>None, no cooking<br>Other
Lighting source	Electricity (mains)<br>Electricity (private generator)<br>Solar energy	Candle<br>Crop residue<br>Firewood<br>Flashlight/Torch<br>Gas lamp<br>Kerosene lamp<br>Other
Floor material	Burnt brick<br>Cement/Concrete<br>Ceramic/Porcelain/Granite/Marble tiles<br>Stone<br>Terrazzo/Terrazzo tiles<br>Vinyl tiles	Earth/Mud<br>Wood<br>Other
Roof material	Cement/Concrete<br>Metal sheet<br>Roofing tile<br>Slate/Asbestos	Bamboo<br>Mud/Mud bricks/Earth<br>Thatch/Palm leaf or Raffia<br>Wood<br>Other
Wall material	Burnt bricks<br>Cement blocks/Concrete<br>Landcrete<br>Metal sheet/Slate/Asbestos<br>Stone	Bamboo<br>Mud brick/Earth<br>Palm leaf/Thatch (grass)/Raffia<br>Wood<br>Other

For peer review only

Supplementary appendix 3. Under-five mortality rates in neighbourhoods of the Greater Accra Metropolitan Area from sensitivity analyses replacing zero MAC-5q0 estimates with **a)** 0.0001 and **b)** the minimum estimated non-zero 5q0 (0.00632). Neighbourhood boundaries are shown in grey, district boundaries are shown in black. The colour scale diverges at the mean under-five mortality rate across all GAMA neighbourhoods. Neighbourhoods with U5M above the GAMA average are shown in orange; neighbourhoods with U5M below the GAMA average are shown in purple.

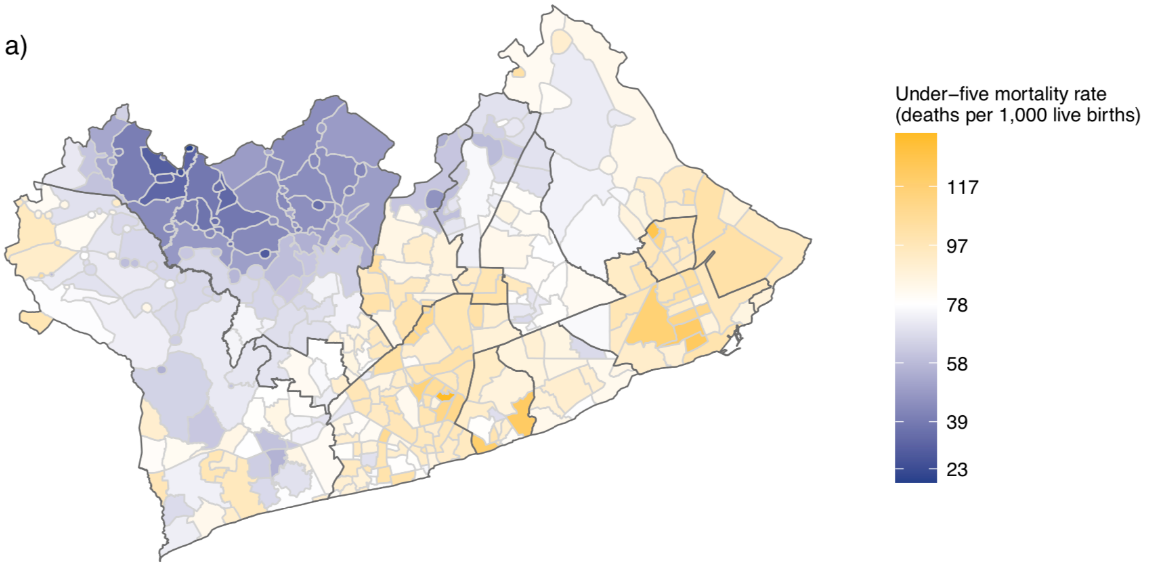

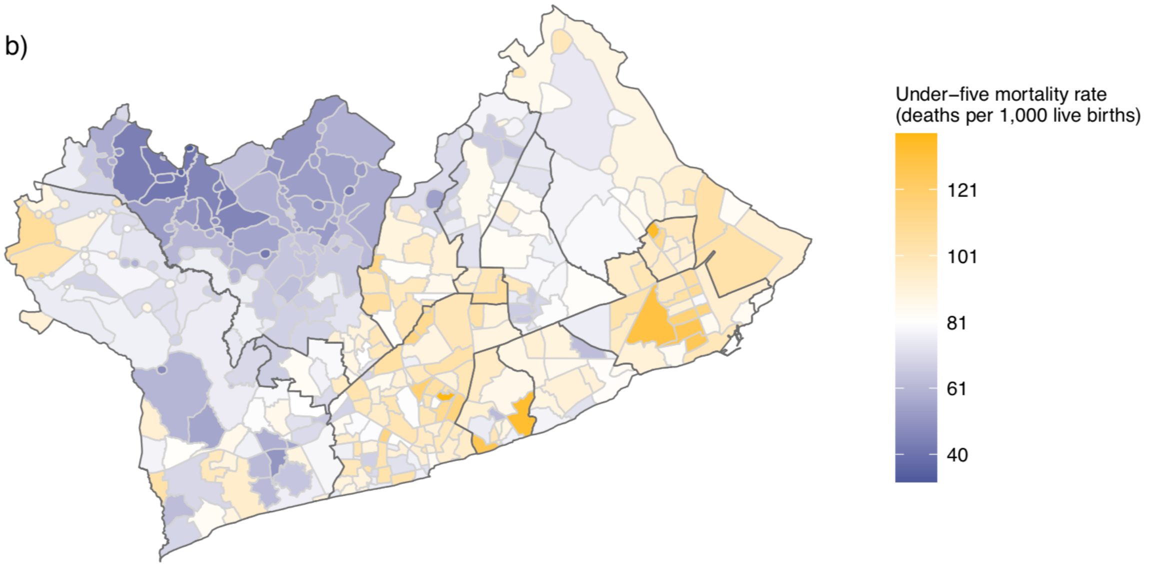

Supplementary appendix 4: The distribution of under-five mortality at increasing levels of neighbourhood living and socio-economic condition indicators.

Neighbourhoods are grouped into quintiles based on the measured indicator, separately by urban or peri-urban area. The horizontal line and box show the median and interquartile range.

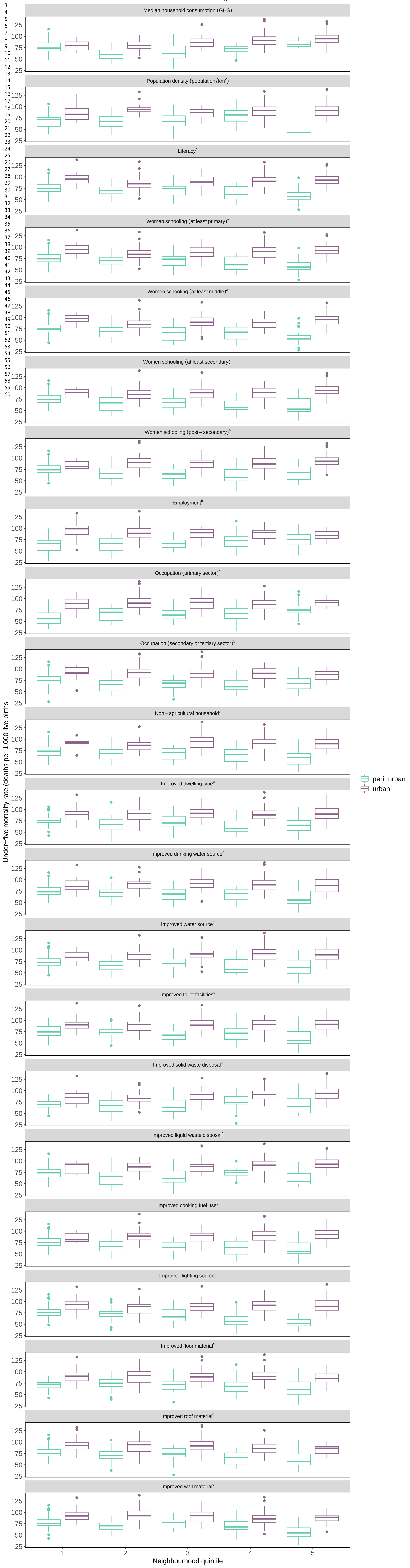

Neighbourhood indicator measured as
(a) the proportion of women of childbearing age;
(b) the proportion of population of working age (15–64 years); and,
(c) the proportion of total population.

BMJ Open

Quantifying within-city inequalities in child mortality across neighbourhoods in Accra, Ghana: A Bayesian spatial analysis.

Journal:	*BMJ Open*
Manuscript ID	bmjopen-2021-054030.R1
Article Type:	Original research
Date Submitted by the Author:	02-Dec-2021
Complete List of Authors:	Bixby, Honor; McGill University,<br>Bennett, James; Imperial College London<br>Bawah, Ayaga; University of Ghana, Regional Institute for Population Studies<br>Arku, Raphael; University of Massachusetts Amherst<br>Annim, Samuel ; Ghana Statistical Service; University of Cape Coast<br>Anum, Jacqueline; Ghana Statistical Service<br>Mintah, Samilia; Ghana Statistical Service<br>Schmidt, Alexandra; McGill University<br>Agyei-Asabere, Charles; University of Ghana<br>Robinson, Brian; McGill University<br>Cavanaugh, Alicia; McGill University<br>Agyei-Mensah, Samuel; University of Ghana<br>Owusu, George; University of Ghana<br>Ezzati, Majid; Imperial College London; University of Ghana<br>Baumgartner, Jill ; McGill University
**Primary Subject Heading**:	Epidemiology
Secondary Subject Heading:	Public health
Keywords:	Community child health < PAEDIATRICS, EPIDEMIOLOGY, PUBLIC HEALTH

SCHOLARONE™
Manuscripts

I, the Submitting Author has the right to grant and does grant on behalf of all authors of the Work (as defined in the below author licence), an exclusive licence and/or a non-exclusive licence for contributions from authors who are: i) UK Crown employees; ii) where BMJ has agreed a CC-BY licence shall apply, and/or iii) in accordance with the terms applicable for US Federal Government officers or employees acting as part of their official duties; on a worldwide, perpetual, irrevocable, royalty-free basis to BMJ Publishing Group Ltd ("BMJ") its licensees and where the relevant Journal is co-owned by BMJ to the co-owners of the Journal, to publish the Work in this journal and any other BMJ products and to exploit all rights, as set out in our licence.

The Submitting Author accepts and understands that any supply made under these terms is made by BMJ to the Submitting Author unless you are acting as an employee on behalf of your employer or a postgraduate student of an affiliated institution which is paying any applicable article publishing charge ("APC") for Open Access articles. Where the Submitting Author wishes to make the Work available on an Open Access basis (and intends to pay the relevant APC), the terms of reuse of such Open Access shall be governed by a Creative Commons licence – details of these licences and which Creative Commons licence will apply to this Work are set out in our licence referred to above.

Other than as permitted in any relevant BMJ Author's Self Archiving Policies, I confirm this Work has not been accepted for publication elsewhere, is not being considered for publication elsewhere and does not duplicate material already published. I confirm all authors consent to publication of this Work and authorise the granting of this licence.

For peer review only

Quantifying within-city inequalities in child mortality across neighbourhoods in Accra, Ghana: A Bayesian spatial analysis.

Honor Bixby[1,2*], James E. Bennett[3,4], Ayaga A. Bawah[5], Raphael E. Arku[6], Samuel K. Annim[7,8], Jacqueline D. Anum[7], Samilia E. Mintah[7], Alexandra M. Schmidt[1], Charles Agyei-Asabere[5], Brian E. Robinson[9], Alicia Cavanaugh[9], Samuel Agyei-Mensah[10], George Owusu[11], Majid Ezzati[3,4,5], Jill Baumgartner[1,2]

[1]Department of Epidemiology, Biostatistics and Occupational Health, McGill University, Montreal, Quebec, Canada.

[2]Institute for Health and Social Policy, McGill University, Montreal, Canada.

[3]Department of Epidemiology and Biostatistics, School of Public Health, Imperial College London, London, UK.

[4]MRC Centre for Environment and Health, Imperial College London, London, UK.

[5]Regional Institute for Population Studies, University of Ghana, Accra, Ghana.

[6]Department of Environmental Health Sciences, University of Massachusetts Amherst, Amherst, Massachusetts, USA.

[7]Ghana Statistical Service, Accra, Ghana.

[8]Department of Applied Economics, University of Cape Coast, Cape Coast, Ghana.

[9]Department of Geography, McGill University, Montreal, Quebec, Canada.

[10]Department of Geography and Resource Development, University of Ghana, Accra, Ghana.

[11]Institute of Statistical, Social and Economic Research, University of Ghana, Accra, Ghana.

*Correspondence to honor.bixby@mcgill.ca

For peer review only

Abstract

Objective Countries in sub-Saharan Africa suffer the highest rates of child mortality worldwide. Urban areas tend to have lower mortality than rural areas, but these comparisons likely mask large within-city inequalities. We aimed to estimate rates of under-five mortality (U5M) at the neighbourhood level for Ghana's Greater Accra Metropolitan Area and measure the extent of intra-urban inequalities.

Methods We accessed data on >700,000 women aged 25-49 years living in the Greater Accra Metropolitan Area (GAMA) using the most recent Ghana census (2010). We summarised counts of child births and deaths by five-year age group of women and neighbourhood (n=406) and applied indirect demographic methods to convert the summaries to yearly probabilities of death before age five years. We fitted a Bayesian spatio-temporal model to the neighbourhood U5M probabilities to obtain estimates for the year 2010, and examined their correlations with indicators of neighbourhood living and socio-economic conditions.

Results U5M varied almost five-fold across neighbourhoods in GAMA in 2010, ranging from 28 (95% credible interval (CrI): 8-63) to 138 (95% CrI: 111-167) deaths per 1,000 live births. U5M was highest in neighbourhoods of the central urban core and industrial areas, with an average of 95 deaths per 1000 live births across these neighbourhoods. Peri-urban neighbourhoods performed better, on average, but rates varied more across neighbourhoods compared to neighbourhoods in the central urban areas. U5M was negatively correlated with multiple indicators of improved living and socio-economic conditions among peri-urban neighbourhoods. Among urban neighbourhoods, correlations with these factors were weaker or, in some cases, reversed, including with median household consumption and women's schooling.

Conclusion Reducing child mortality in high-burden urban neighbourhoods in GAMA, where a substantial portion of the urban population resides, should be prioritised as part of continued

For peer review only

efforts to meet the Sustainable Development Goal national target of less than 25 deaths per 1,000 live births.

For peer review only

For peer review only - http://bmjopen.bmj.com/site/about/guidelines.xhtml

Strength and limitations of this study

- We accessed the full microdata of latest census (2010) in Ghana that contained birth history data for over 700,000 women aged 25-49 years living in the Greater Accra Metropolitan Area.
- Georeferenced census data allowed estimation of under-five mortality at the fine spatial scale of neighbourhood with full coverage of the Greater Accra Metropolitan Area.
- We used a flexible Bayesian spatial-temporal model that allowed each neighbourhood's mortality estimate to be informed by its own data and that of surrounding neighbourhoods and incorporated weights for the number of births recorded.
- Under-five mortality was indirectly calculated from the summary birth history data because complete birth and death registration data were unavailable. These data may be subject to recall errors.
- No data were available on cause of death.

For peer review only

Background

Recent decades have delivered marked reductions in child mortality across all world regions.[1] Despite improvements, almost eight percent of all deaths globally in 2019 were children under five years of age,[2] mostly due to preventable and treatable causes linked to infection and malnutrition.[3] The rate of under-five mortality (U5M) in sub-Saharan Africa SSA far exceeds other regions, estimated at 76 deaths per 1,000 live births in 2019, and under-five deaths in the region account for a growing proportion of the global total.[1]

On average, children living in cities across low- and middle-income regions have a survival advantage over their rural counterparts, largely due to improved education, employment and healthcare opportunities in urban areas.[4-6] While still predominantly rural, SSA has the world's fastest growing urban population and cities are expected to absorb over 75% of the region's population growth over the next three decades.[7] This offers many opportunities for continued improvement in child mortality, however, infrastructure and basic service provision, remain major challenges for cities in the region, exacerbated by the rapid pace of urban population growth. Over half of the urban population - more than in any other world region - lives in slums and informal settlements that are often characterised by poverty and concentrated deprivation.[8]

The social determinants of health refer to the conditions in which people are born, grow, live, work and age and have important influence on health inequities.[9 10] Social gradients in the health of children are well-documented, whereby children born into deprivation have lower chances of survival and prosperity.[9-11] In cities, health outcomes and their social, economic and environmental determinants can vary dramatically between households and neighbourhoods.[12 13] The mortality gap between children living in slum versus non-slum urban areas in SSA, for example, can be as large as the gap between rural and urban children.[6 14-16] Inadequate housing,

For peer review only - http://bmjopen.bmj.com/site/about/guidelines.xhtml

electricity and clean fuel access, water and sanitation facilities, nutrition and healthcare services are among the pathways through which income or education levels, among other factors termed 'social stratifiers',[9] can increase the susceptibility or hazardous exposures of those most deprived. These mechanisms can act at the individual and household level or area level, whereby people with the fewest means are spatially sorted into neighbourhoods with the poorest infrastructure, known as segregation.[17 18] This in turn can contribute to intra-urban health inequalities, including in child mortality, seen at the small area level, where advantage tends to cluster.[13 16 19 20]

There is substantial evidence that health outcomes vary at small spatial scales[21-26] and local neighbourhood factors are increasingly recognised as important drivers of population health inequalities.[21 27-30] Subnational estimates of child mortality are often at too course of a spatial scale to capture local variation across cities.[22 31 32] Increased knowledge of the spatial heterogeneity of child mortality within cities at finer scales in SSA countries is necessary to target interventions and programs towards high risk populations, accelerate the progress necessary to reach the United Nations' Sustainable Development Goal (SDG) target of less than 25 deaths per 1,000 live births for all countries by 2030 (Goal 3, Target 3.2),[33] and better understand the determinants of such inequalities.[34]

With unique access to the complete records of the most recent Ghana Population and Housing Census,[35] we aimed to estimate rates of under-five mortality (U5M) at the neighbourhood level across the Greater Accra Metropolitan Area (GAMA), providing insight into the magnitude of intra-urban inequalities in child mortality within a rapidly growing, low-middle income city. We quantified under-five mortality rates for 2010, the year of the census, and, aligned with previous studies using census data, examined their relationships with neighbourhood-level

For peer review only - http://bmjopen.bmj.com/site/about/guidelines.xhtml

indicators of socioeconomic and living conditions.[21 26 28] This study was conducted within the Pathways to Equitable Healthy Cities study (http://equitablehealthycities.org/).

Methods

Study setting

Ghana is among the most urbanised countries in SSA with an estimated urban population of over 18 million (58% of the total population) that is growing by ~3% each year.[7] GAMA is Ghana's administrative and economic capital and accounted for 29% of the country's urban population in 2010.[35] It covers ~1500 km^2 on the southern coast of the Greater Accra region. According to the 2010 census, GAMA comprises 5019 census enumeration areas (EAs) – the smallest administrative geographical unit in Ghana – nested within 406 localities and 12 districts or 'municipalities' (**Figure 1)**.

The centrally located Accra Metropolitan Area (AMA) - together with the more heavily industrialised Tema and Ashaiman municipalities to the east - contain the most densely populated neighbourhoods. AMA contains the central business district and functions as the city's commercial, industrial and administrative centre.[36 37] Rapid development since Ghana's independence in 1957 has contributed to increasing congestion in AMA's residential areas. Planned residential neighbourhoods in AMA remain as legacies of the colonial era, while migrants and low-income individuals have been pushed into slums and other low-income neighbourhoods lacking basic services and infrastructure.[17] Tema is GAMA's planned industrial hub with structured housing developments and services, and was the fastest growing municipality following independence. A small fraction (~5%) of GAMA's population lives in areas classified in the 2010 census as rural, mostly in northern GAMA and predominantly in the Ga West and Ga South districts. These districts are characterised by sprawling urban

For peer review only - http://bmjopen.bmj.com/site/about/guidelines.xhtml

development with high rates of population growth since the 1970s due to congestion of the city centre.[36 38]

Overall the under-five mortality rate in Ghana almost halved from 1990 to 2010, though considerable subnational inequality persisted.[1 31 32] During this period, the government implemented several national health policies and programs to improve the use and delivery of maternal and child health care services.[39] The National Health Insurance Scheme provides free health care to participants[40] and, since 2008, enrolment is free of charge for pregnant women and children under 18 years of age.[41] Ante- and postnatal visits, facility delivery (including emergency obstetric care) and neonatal care are all included under the scheme. The User Fees Exemption for Delivery Care was scaled up in 2005 and exempts pregnant women who are not enrolled in NHIS from paying delivery fees.[42] Ghana's 2007-2015 Child Health Policy aimed to unify fragmented program delivery under a recommended continuum of care for mothers and children. scaling up interventions with proven efficacy to prevent child deaths, including, for example, oral rehydration therapy and zinc for treatment of diarrhoea, vitamin A supplementation and antibiotic treatment for pneumonia.[43] Together, these efforts have contributed to reductions in overall child mortality and in inequalities between subregions, though wider concerns have persisted over the quality of care.[44] In Accra, women enrolled in NHIS were more likely to seek formal care and visit clinics but enrolment rates were lower (under 35%) among women of child-bearing age compared with women over the age of 50.[41] Although most (>90%) births in Greater Accra take place in a health facility in the presence of a skilled health professional, the coverage rate of age-appropriate vaccines in children drops with age (from 76% to 48% comparing children aged 1 versus children aged 2-3 years, respectively). This indicates considerable variability in continued access to care for children.[45]

For peer review only - http://bmjopen.bmj.com/site/about/guidelines.xhtml

Data

We accessed the full microdata of the most recent Ghana Population and Housing Census, conducted in 2010, via the Ghana Statistical Service. The census collected information on the number of children born and surviving at the time of survey, known as their summary birth history, for all females aged 12 years and older. Other individual and household characteristics captured in the census include employment status, occupational industry, schooling level, literacy, household amenities (including type of cooking fuel, drinking and non-drinking water source, sanitation facilities and waste disposal in use), dwelling type and structural features (including roof, floor, and wall materials), and EA of residence. Together, these provide information on the socioeconomic and living environments.

We obtained a shapefile from the Ghana Statistical Service with all GAMA EAs, localities and districts geocoded according to the 2010 census geographies. Localities within GAMA were the neighbourhood units used in our analysis, each containing between one and 95 EAs. We linked the census data and the shapefile using codes that uniquely identified EAs to determine each individual's neighbourhood of residence. Neighbourhoods were defined by the Ghana Statistical Service and are the administrative units at which urban versus rural classification is defined in Ghana; those with 5,000 inhabitant or more are considered urban, and rural otherwise. Some neighbourhoods were recently sub-divided due to population growth and thus did not meet the urban population threshold despite the urban designation of their constituent EAs in the census. We therefore classified GAMA neighbourhoods as urban or peri-urban according to the historic census-derived urban-rural designation of their constituent EAs. Most neighbourhoods (98%) comprised exclusively urban or exclusively rural EAs. We classified neighbourhoods that contained both urban and rural EAs as urban if over 50% of the population lived in urban EAs, and peri-urban otherwise (**Figure 1**). Ghana does not have an official

For peer review only - http://bmjopen.bmj.com/site/about/guidelines.xhtml

definition that distinguishes peri-urban from rural neighbourhoods; however, we used the term peri-urban to better describe 'rural' neighbourhoods that are located within the administrative border of the Greater Accra Metropolitan Area on the periphery of the densely populated inner-city and industrial areas (**Figure 1**).

To assess U5M, we summarised the birth history data of women of reproductive age (15-49 years) by five-year age group and neighbourhood. The Maternal Age Cohort (MAC) method was used to estimate the neighbourhood probability of death for children before the age of five (5q0) for each five-year age group, based on the number of children ever born, proportion of children who have died and average parity.[46] The MAC method outperforms alternative methods for estimating U5M from summary birth history data for subnational populations.[47] Each 5q0 was assigned to a reference year prior to the census, using maternal age as a proxy measure for duration of exposure to risk of death for a child. The assigned 5q0 reference years covered the period from 1990 to 2005. We excluded 5q0 estimates derived from women aged 15-19 and 20-24 years owing to the low numbers of births recorded for these age groups in many neighbourhoods, which could lead to spurious fluctuations in the 5q0 estimates, especially in the more sparsely populated peri-urban areas. Notably, this is common practice when using demographic methods to estimate population under-five mortality rates.[46-48] This left five 5q0 estimates for each neighbourhood (one derived from each five-year age group of women aged 25-49 years). The assigned 5q0 reference years covered the period from 1990 to 2005.

Statistical analysis

To obtain neighbourhood estimates of 5q0 for 2010, the year of the census, we fitted a Bayesian space-time model to the MAC-derived 5q0 estimates across all 2,030 neighbourhood-reference

For peer review only - http://bmjopen.bmj.com/site/about/guidelines.xhtml

year units, transformed to the probit scale. The model included a linear time trend that could vary by neighbourhood. The time trend allowed data from different reference years, each of which is associated with a different age group, to inform the 5q0 in 2010. The neighbourhood intercepts and slopes were modelled using the Besag, York, and Mollié model,[49] where information is shared locally (i.e., amongst adjacent neighbourhoods) through spatially structured random effects with a conditional autoregressive prior, and globally through spatially unstructured Gaussian random effects. Neighbourhood-specific intercept and slope values were estimated by the sum of their respective spatially structured and spatially unstructured random effects. The prior distributions in the Bayesian framework allow the neighbourhood-specific parameters to be estimated by a neighbourhood's own data and data of contiguous neighbourhoods. This approach balances between overly unstable within-neighbourhood estimates and overly simplified aggregate estimates for all of GAMA. The reported estimate for the wealthy Ringway neighbourhood in AMA is informed entirely by data in bordering neighbourhoods as the data on child deaths were considered implausible (see **Supplementary Appendix 1**). To account for excess variability resulting from small numbers of children born to women in a given age-group and neighbourhood, we included a weighted variance term that gave more weight to estimates derived from a higher number of births. Samples from the posterior distributions of the intercepts and slopes were used to estimate 5q0 for the year 2010.

To avoid infinite values on the probit scale, we adjusted all MAC-derived 5q0 estimates of zero (n=146; 7%) to half the minimum estimated non-zero value across all units (0.00316). We conducted sensitivity analyses to ensure our results were robust to this choice by replacing zero estimates with a lower value of 0.0001 and with the minimum estimated non-zero 5q0.

For peer review only - http://bmjopen.bmj.com/site/about/guidelines.xhtml

We monitored convergence using trace plots and obtained 5000 post burn-in samples from the posterior distributions of model parameters. We summarised the distributions of neighbourhood-specific parameters to report neighbourhood U5M estimates for 2010 and mean U5M across neighbourhoods within districts, with 95% credible intervals (CrI) that represent the mean and the 2.5th and 97.5th percentiles of posterior samples, respectively. We present neighbourhood U5M estimates as deaths per 1,000 live births.

We calculated neighbourhood-level summary statistics of the individual and household characteristics within each neighbourhood to provide context for our mortality results. We used the within-neighbourhood median household consumption as a measure of neighbourhood socioeconomic level. Household consumption is considered a better indicator of living standards than household income in low- and middle-income settings[50 51] The census did not include consumption data, so we used small-area estimation methods to indirectly calculate consumption based on household characteristics described in detail elsewhere.[52] Briefly, we used the 2012 Ghana Living Standards Survey to develop a statistical relationship between household characteristics and consumption. Then using those same household characteristics, we predicted consumption for households in the census. We additionally calculated population density; the proportion of the women of reproductive age (15-49 years) who were literate, had schooling to at least primary, middle, secondary and postsecondary levels; the proportion of the working age (15-64 years) population in any employment and in primary, secondary and tertiary sector occupations; and the proportion of households with indicators of improved living conditions (including dwelling type, materials of flooring, roofing and walls, methods of solid and liquid disposal, type of toilet facility, type of cooking fuel and type of drinking and other water source). Details of our classification of the census responses into indicators of 'improved' versus 'unimproved' living conditions are provided in the **Supplementary Appendix 2**.

For peer review only

We measured the correlations between neighbourhood U5M and neighbourhood socioeconomic and living environment indicators using the non-parametric Spearman's rank method. We measured the correlations across all GAMA neighbourhoods, and separately across urban and peri-urban neighbourhoods.

All analyses were implemented in the open-source statistical software R version 3.6.1. The Bayesian model was implemented using the NIMBLE package version 0.9.1.

Patient and Public Involvement

The study used secondary data only.

Results

There were 713,581 women aged 25-49 years living in GAMA in 2010, who reported a total of 1,425,008 children, 1,312,030 (92.1%) of whom were alive at the time of the census. The number of women in this age range per neighbourhood ranged from 15 to 16,061 (median = 564). The proportion of their children born who had died ranged from 0 to 20% across neighbourhoods.

In 2010, the mean neighbourhood U5M rate in GAMA was 80 deaths per 1,000 live births (95% CrI 76-84). This compares to an estimated 69 deaths per 1000 live births nationally in Ghana.[1] Across all of GAMA, neighbourhood U5M varied almost five-fold, ranging from 28 deaths per 1,000 live births (95% CrI 8-63) in the Fantsenkor neighbourhood located in the north of GAMA to 138 deaths per 1,000 live births (95% CrI 111-167) in the Mamobi neighbourhood in the city's urban core (**Figure 2a and Figure 3**). The variation was higher

For peer review only - http://bmjopen.bmj.com/site/about/guidelines.xhtml

across peri-urban neighbourhoods compared to across urban neighbourhoods, ranging from 28 to 116 versus from 52 to 137 deaths per 1,000 live births.

We found substantial variation in U5M between and within GAMA's twelve districts. The within-district mean neighbourhood U5M was highest at 101 deaths per 1,000 live births in the Tema (95% CrI 89-113) and Ashaiman (95% CrI 89-114) municipalities situated in the southeast and east of GAMA, respectively. Both municipalities comprised only urban neighbourhoods. The within-district mean neighbourhood U5M was lowest in the northern Ga West district (60 deaths per 1,000 live births, 95% CrI 52-68), where over 70% of neighbourhoods were considered peri-urban (**Figure 2a**). Although Ga West had the lowest mean mortality among GAMA districts, it had the highest within-district inequality (measured as the relative difference between the neighbourhood with the highest versus lowest mortality), with a 3.8-fold difference between the worst and best performing neighbourhoods. Within-district inequality was lowest in the Ga Central and Kpone Katamanso municipalities (1.4-fold difference across neighbourhoods).

The ten percent of neighbourhoods with the lowest U5M were concentrated in just three neighbouring districts in western GAMA, namely Ga West, Ga East and Ga South. The ten percent of neighbourhoods with the highest U5M were more dispersed, present in eight of the twelve GAMA districts, though almost half of highest mortality neighbourhoods fell within the urban core of AMA (**Figure 2b**). Neighbourhood mortality was consistently high in AMA where 80% of neighbourhoods (n=64) had U5M rates higher than the mean across GAMA neighbourhoods. This was true also for the Tema and Ashaiman municipalities, where 95% and 100% of neighbourhoods, respectively, had higher than average under-five mortality (**Figure 3**). The spatial pattern of U5M reported were unchanged in our sensitivity analyses

For peer review only - http://bmjopen.bmj.com/site/about/guidelines.xhtml

(**Supplementary Appendix 3**). The mean absolute difference in neighbourhood U5M estimates was 2.24 deaths per 1000 live births in our analysis replacing zero estimates of 5q0 with 0.0001, and -0.73 in our analysis replacing zero estimates with the minimum estimated non-zero 5q0 value.

The correlations between neighbourhood U5M and indicators of improved living and socio-economic conditions in peri-urban areas were distinct from urban neighbourhoods located mostly in the urban core and industrial areas (**Table 1**). In peri-urban areas, U5M was inversely correlated with most of the indicators of improved neighbourhood conditions analysed. The strongest associations were with the proportions of neighbourhood residents living in houses built with improved wall and roof materials, and with an improved lighting source, followed by the share of women aged 15-49 with at least primary or middle education or who were literate (Spearman's rho: -0.41 to -0.50). By comparison, correlations were weaker or reversed in urban neighbourhoods. Similar inverse correlations, though smaller in magnitude (Spearman's rho: -0.14 to -0.23), were found with the share of working age population engaged in secondary or tertiary sector occupations and the proportion of residents living in housing with improved wall and roof materials. No relationship was found for several indicators, including levels of women's literacy and access to improved drinking water source (Spearman's rho: 0.12 and -0.02, respectively). For others, there was a positive correlation, most notably with indicators of socioeconomic status, e.g., share of women educated to middle school level or above and average household consumption (Spearman's rho: 0.14 to 0.35).

Discussion

Our high spatial resolution analysis of child mortality in GAMA, one of the largest metropolitan areas in SSA, revealed considerable intra-urban inequality in U5M with the

For peer review only - http://bmjopen.bmj.com/site/about/guidelines.xhtml

highest levels in neighbourhoods located in the city's urban core and in more heavily industrial areas. Notably, even GAMA's lowest mortality neighbourhood had a mortality rate equivalent to the Dominican Republic and Bhutan which ranked 129th and 130th , respectively, among countries and territories worldwide in the UN's most recent estimates.[1] At present, no country has an estimated average child mortality rate as high as GAMA's highest mortality neighbourhoods.

A general pattern of higher child mortality extended across inner city and more industrial neighbourhoods, despite higher median household consumption and generally higher levels of post-primary education among women in these neighbourhoods relative to their peri-urban counterparts. Moreover, child mortality tended to be higher in better-off neighbourhoods of urban GAMA. The spatial concentration of people across a wide socio-economic spectrum may generate what has been termed 'negative health externalities',[13] whereby localised inequalities compromise the real or perceived capacity of low-resourced individuals to sustain good health. Households that are better off may be negatively impacted by environmental degradation that is not bounded by household or neighbourhood boundaries, e.g., poor community sanitation or air pollution from neighbours' solid waste burning or household use of solid fuel stoves.[53-55] It is important to highlight, however, that we cannot draw firm conclusions regarding the harmful impact of localised socio-economic inequalities on the health of individuals within neighbourhoods from these observed correlations in our study. Associations at the area level that include neighbourhoods with diverse populations may simply obscure any association between wealth and health operating at the individual or household level.

For peer review only

Child mortality can be seen in some places to follow historical patterns of socio-economic segregation in central Accra.[17] The wealthier Cantonments neighbourhood – home to European settlers in the colonial era – was among the pockets of lower-than-average U5M (63 deaths per 1,000 live births), contrasted with higher rates found in low-income or 'slum' neighbourhoods, including Mamobi, Nima and New Town (83-138 deaths per 1,000 live births). However, other low-income neighbourhoods had U5M rates below the GAMA average, e.g., Jamestown and Sabon Zongo. The health penalty of areas with concentrated poor living conditions that arise from rapid, unplanned population growth, compiled by underinvestment in public services and infrastructure, in SSA cities is well established and supported by previous studies conducted in central Accra and Nairobi.[6 14 56-59] The high mortality rates in city slums in Kenya contributed to stalled progress in urban compared with rural areas .[59] Many low-income neighbourhoods in Accra have historically comprised mostly native Ga people and African migrants.[20] The variation seen across poor areas suggests distinct characteristics of these neighbourhoods and vulnerabilities of the people living in them.[53 60-63] Lower U5M rates in some of these neighbourhoods may reflect the success of targeted interventions to reduce mortality in the poorest communities.[64]

We observed the lowest rates of U5M in peri-urban neighbourhoods, where residents can still benefit from the urban services that contribute to improved child survival in cities – most births in Greater Accra, for example, take place in a health facility in the presence of a skilled health professional[45] – but avoid the potential hazards of inner-city living. A healthy selection effect may also contribute to the observed pattern of U5M where recent development of peri-urban neighbourhoods has encouraged wealthier residents to move out of the inner-city in search of better standards of living. Some of the areas considered peri-urban in the Ga districts are more recently developed neighbourhoods mostly occupied by middle- and high-income individuals.

For peer review only - http://bmjopen.bmj.com/site/about/guidelines.xhtml

Our finding of lower U5M in neighbourhoods with improved socio-economic and living conditions in the peri-urban area is consistent with the established social gradient in child mortality,[9-11] and with evidence linking the neighbourhood environment to population health inequalities.[21 27-30] It may reflect greater access to services including health care for women in these neighbourhoods, which can influence the health of mothers and their children. However, these reported ecological correlations only provide broader context to the observed spatial patterns of U5M across GAMA and should not be interpreted as causal.

A notable strength of our study is the fine spatial resolution of our U5M estimates, enabled by access to the full microdata of the 2010 census, including birth histories of over 700,000 women. Previous knowledge of child mortality inequalities in the metropolitan area was limited to broad district aggregates[31 32] or smaller areas in the urban core,[61 65] thereby excluding almost half of GAMA's total population, including residents of the rapidly developing Ga districts and the industrial Tema area.[66] In the absence of complete birth and death registration, summary birth history data used in our study can produce robust estimates of U5M[67] that are comparable to estimates directly calculated from complete birth history data that require detailed questionnaires.[47 68] The ease of summary birth history collection allows for its inclusion in national censuses and, in turn, analysis of U5M at finer spatial scales than is achievable through sample surveys.[69] Our use of census data also enabled straightforward linkage of mortality outcomes with data on socio-economic and living conditions at a common, local spatial scale and avoided issues of sample representativeness. Specifically, the data analysed were of all women aged 25-49 present in GAMA at the time of enumeration, and included those living in regular housing (n=696,279), homeless women/outdoor sleepers (n=13,950), women who were in schools, hospitals, army and service barracks and prisons (n=2,361) and a small proportion from other locations (n=991). People were not excluded from

For peer review only - http://bmjopen.bmj.com/site/about/guidelines.xhtml

For peer review only

the census or this analysis based on migration status. Our analysis makes important methodological advancements from available neighbourhood-level U5M estimates in GAMA.[61 65] Firstly we used a validated method for the use of summary birth history data in U5M estimation.[46 47] Secondly, we applied established Bayesian methods widely used in small area estimation[49] that allow sharing of information across spatial units to make robust estimates for our study neighbourhoods.

Our results show a different spatial pattern of U5M across GAMA compared with previous estimates reported at the district level that indicated higher U5M in the largely peri-urban Ga West district and minimal variability across the rest of GAMA.[31] This inconsistency could be due to a scarcity of data from areas outside the urban core used in the previous analysis. Their results rely heavily on data from the Demographic and Health Surveys that, for example in 2008, sampled 41 women living in only four EAs in Ga West compared with 299 women living in 38 EAs in the AMA. Estimates for Ga West would therefore be informed by data with limited geographical coverage within the district and data from bordering rural areas, where child mortality is on average higher.[6] By comparison, our study included data on children's births and deaths from 41,270 women living in all 92 neighbourhoods of GA West.

Our study has several limitations to be considered for future studies. Changes in GAMA over the last decade may mean our results do not reflect current patterns of U5M. Data from more recent sample surveys, however, lack the coverage, scope and sample sizes of the census which enabled us to conduct our analysis at very high spatial resolution. The georeferenced data in the 2021 Ghana census, which is currently in progress, can be used to provide up-to-date estimates of U5M within GAMA and examine changes since 2010. As is the case for all studies of child mortality using census or sample survey data, we were limited to data on

For peer review only - http://bmjopen.bmj.com/site/about/guidelines.xhtml
For peer review only

neighbourhood of residence at the time of the census and could not account for re-location since the death of a child. We also acknowledge that intervention efforts to prevent child mortality would benefit from more detailed information on the underlying causes of these deaths and age at which GAMA children are most vulnerable.[70 71] In our study, we were unable to separately estimate neonatal, infant, or cause-specific mortality rates with the available methods for mortality estimation from summary birth history data and are unaware of any other data that is representative at the neighbourhood level and would enable this analysis. Investment in national civil registration systems across SSA, including in Ghana, could provide these data in real-time and enable governments to monitor changes and progress in lowering child mortality and reducing inequalities.[72 73] Finally, we highlight that although we report under-five mortality at a fine-spatial scale, populations within neighbourhoods may still be heterogenous in their child mortality risk and its determinants, particularly within the more populous inner city neighbourhoods. This is often discussed in the context of the Modifiable Areal Unit Problem, a statistical bias that can arise when area-level measurements are sensitive to the scale or zoning scheme used.[74]

Conclusions

Our city-scale results contribute to a small but growing number of studies showing that within-city variation child mortality in SSA can be as large as the difference between urban and rural areas or between countries.[6 14 16] Global targets for child mortality, including SDG 3.2, and related monitoring efforts focus entirely on national mortality rates.[33 75] While important for benchmarking countries' overall performance, progress towards SDG 3.2 does not automatically benefit high mortality and vulnerable population sub-groups within countries.[31 76] The heterogeneity of under-five mortality that we found across GAMA neighbourhoods motivates more localised and increasingly disaggregated information on child deaths in order

For peer review only

to deliver effective interventions and continue progress toward meeting national and international targets in a region with relatively limited public health resources.

The determinants of child mortality are multi-faceted, operate at multiple levels and can also interact with one another. As Ghana continues to urbanise, its cities and metropolitan areas, including GAMA, will play an increasingly important role in building on national child survival efforts. The heterogeneity in child mortality across Accra's neighbourhoods highlights the need for an explicit focus on equity in the context of rapid urbanisation in SSA, with an emphasis on the social determinants of health. Concentrated deprivation in households and neighbourhoods can compound the risk of child mortality. Our study identified neighbourhoods with high child mortality in GAMA's central and industrial areas, where the urban poor may still face financial and physical barriers to accessing health services,[41 77] potentially compounding health risks associated with disadvantaged social and living conditions. Universal access to high quality healthcare services can mitigate mortality inequalities in settings where children are born into different socio-economic and environmental circumstances.[70 78 79] There are proven, scalable healthcare interventions that reduce under-five mortality, the causes of which are often preventable and/or treatable.[70 80] In many LMICs the rise in facility births has not produced the expected improvements in child mortality, demonstrating the importance of continued access to quality care throughout the early years of life for all children and mothers across the socio-economic spectrum.[78 81]

Child mortality in peri-urban neighbourhoods was on average lower than inner-city and industrial areas, but it was also more variable and inversely correlated with characteristics that indicate improved socio-economic and living environments of neighbourhoods. Complementary investment in developing infrastructure and services in neighbourhoods

For peer review only - http://bmjopen.bmj.com/site/about/guidelines.xhtml

outside of the urban core, while ensuring that conditions in densely populated central and industrial areas do not deteriorate, could further contribute to improving child mortality and promoting health equity.

For peer review only

For peer review only - http://bmjopen.bmj.com/site/about/guidelines.xhtml

Contributions

HB, ME and JB developed the study concept. HB, AAB, SKA, JDA, SEM, REA, AC and SAM contributed to data collation. HB conducted the statistical analysis with input from JEB, REA, AS, BER, ME and JB. HB, AB, REA, SAM, BER, ME and JB contributed to data interpretation. HB wrote the first draft of the manuscript with input from JB. All authors contributed to revising and finalising the manuscript.

We also thank Tzu-Wei Joy Tseng and Emmalin Buajitti for their help in preparation of the manuscript and helpful discussions during revisions.

Ethics statement

This study was exempt from ethical approval because it used anonymised secondary data.

Funding

This work is supported by the Pathways to Equitable Healthy Cities grant from the Wellcome Trust [209376/Z/17/Z]. HB is supported by a Canadian Institute of Health Research Banting Postdoctoral Fellowship. For the purpose of Open Access, the author has applied a CC BY public copyright licence to any Author Accepted Manuscript version arising from this submission. The funders had no role in the study design, data collection and analysis, decision to publish, or preparation of the manuscript.

Competing interests

None to declare.

Data availability

Estimates of neighbourhood U5M will be made available at http://equitablehealthycities.org/data-download/. The full microdata of the 2010 Ghana Population and Housing Census are with the Ghana Statistical Service and are not publicly available. Data from a random 10% sample of households enumerated in the 2010 Population and Housing census are publicly available and can be downloaded from Ghana Statistical Services online data catalogue (https://www2.statsghana.gov.gh/nada/index.php/catalog/51).

Code availability

The Bayesian model code will be made available at http://equitablehealthycities.org/data-download/.

For peer review only

References

1. United Nations Inter-agency Group for Child Mortality Estimation. Levels & Trends in Child Mortality. New York: UN, 2020.
2. Wang H, Abbas KM, Abbasifard M, et al. Global age-sex-specific fertility, mortality, healthy life expectancy (HALE), and population estimates in 204 countries and territories, 1950–2019: a comprehensive demographic analysis for the Global Burden of Disease Study 2019. *Lancet* 2020;396(10258):1160-203. doi: 10.1016/S0140-6736(20)30977-6
3. GBD 2015 Child Mortality Collaborators. Global, regional, national, and selected subnational levels of stillbirths, neonatal, infant, and under-5 mortality, 1980-2015: a systematic analysis for the Global Burden of Disease Study 2015. *Lancet* 2016;388(10053):1725-74. doi: 10.1016/S0140-6736(16)31575-6
4. Balk D, Pullum T, Storeygard A, et al. A spatial analysis of childhood mortality in West Africa. *Popul Space Place* 2004;10(3):175-216. doi: 10.1002/psp.328
5. Corker J. Fertility and child mortality in urban West Africa: leveraging geo-referenced data to move beyond the urban/rural dichotomy. *Popul Space Place* 2017;23(3):e2009. doi: 10.1002/psp.2009 [published Online First: 2017/09/26]
6. Fink G, Günther I, Hill K. Slum residence and child health in developing countries. *Demography* 2014;51(4):1175-97. doi: 10.1007/s13524-014-0302-0 [published Online First: 2014/06/05]
7. United Nations Department of Economic and Social Affairs PD. World urbanization prospects, 2018 revision. New York: UN, 2019.
8. UN-Habitat. World Cities Report 2020, 2020.
9. Commission on Social Determinants of Health. Closing the gap in a generation: health equity through action on the social determinants of health: final report of the commission on social determinants of health: World Health Organization 2008.
10. Marmot M. Social determinants of health inequalities. *Lancet* 2005;365(9464):1099-104.
11. Chao F, You D, Pedersen J, et al. National and regional under-5 mortality rate by economic status for low-income and middle-income countries: a systematic assessment. *Lancet Glob Health* 2018;6(5):e535-e47. doi: 10.1016/S2214-109X(18)30059-7
12. Ezzati M, Webster CJ, Doyle YG, et al. Cities for global health. *Bmj* 2018;363:k3794. doi: 10.1136/bmj.k3794 [published Online First: 2018/10/05]
13. Montgomery M, Hewett PC. Urban poverty and health in developing countries: household and neighborhood effects. *Demography* 2005;42(3):397–425. doi: 10.1353/dem.2005.0020
14. Günther I, Harttgen K. Deadly cities? Spatial inequalities in mortality in sub-Saharan Africa. *Popul Dev Rev* 2012;38(3):469-86. doi: 10.1111/j.1728-4457.2012.00512.x
15. Kyu HH, Shannon HS, Georgiades K, et al. Association of urban slum residency with infant mortality and child stunting in low and middle income countries. *Biomed Res Int* 2013;2013:604974. doi: 10.1155/2013/604974
16. Timæus IM, Lush L. Intra-urban differentials in child health. *Health Transit Rev* 1995;5(2):163-90.
17. Agyei-Mensah S, Owusu G. Segregated by neighbourhoods? A portrait of ethnic diversity in the neighbourhoods of the Accra Metropolitan Area, Ghana. *Popul Space Place* 2010;16(6):499-516.
18. Musterd S. Handbook of urban segregation: Edward Elgar Publishing 2020.
19. Elliott P, Wartenberg D. Spatial epidemiology: current approaches and future challenges. *Environmental health perspectives* 2004;112(9):998-1006. doi: 10.1289/ehp.6735 [published Online First: 2004/06/17]

For peer review only - http://bmjopen.bmj.com/site/about/guidelines.xhtml

20. Weeks JR, Hill AG, Getis A, et al. Ethnic residential patterns as predictors of intra-urban child mortality inequality in Accra, Ghana. *Urban Geogr* 2006;27(6):526-48. doi: 10.2747/0272-3638.27.6.526 [published Online First: 2006/01/01]
21. Boing AF, Boing AC, Cordes J, et al. Quantifying and explaining variation in life expectancy at census tract, county, and state levels in the United States. *Proc Natl Acad Sci* 2020;117(30):17688. doi: 10.1073/pnas.2003719117
22. Dwyer-Lindgren L, Squires ER, Teeple S, et al. Small area estimation of under-5 mortality in Bangladesh, Cameroon, Chad, Mozambique, Uganda, and Zambia using spatially misaligned data. *Popul Health Metr* 2018;16(1):13. doi: 10.1186/s12963-018-0171-7
23. Dwyer-Lindgren L, Stubbs RW, Bertozzi-Villa A, et al. Variation in life expectancy and mortality by cause among neighbourhoods in King County, WA, USA, 1990–2014: a census tract-level analysis for the Global Burden of Disease Study 2015. *Lancet Public Health* 2017;2(9):e400-e10. doi: https://doi.org/10.1016/S2468-2667(17)30165-2
24. Rashid T, Bennett JE, Paciorek CJ, et al. Life expectancy and risk of death in 6791 communities in England from 2002 to 2019: high-resolution spatiotemporal analysis of civil registration data. *Lancet Public Health* 2021;6(11):e805-e16. doi: 10.1016/S2468-2667(21)00205-X
25. Monnat SM, Peters DJ, Berg MT, et al. Using census data to understand county-level differences in overall drug mortality and opioid-related mortality by opioid type. *Am J Public Health* 2019;109(8):1084-91. doi: 10.2105/AJPH.2019.305136
26. Nakaya T, Honjo K, Hanibuchi T, et al. Associations of all-cause mortality with census-based neighbourhood deprivation and population density in Japan: a multilevel survival analysis. *PLoS One* 2014;9(6):e97802. doi: 10.1371/journal.pone.0097802
27. Diez Roux AV. Neighborhoods and health: What do we know? What should we do? . *Am J Public Health* 2016;106(3):430-31. doi: 10.2105/ajph.2016.303064
28. Borrell LN, Diez Roux AV, Rose K, et al. Neighbourhood characteristics and mortality in the Atherosclerosis Risk in Communities Study. *Int J Epidemiol* 2004;33(2):398-407. doi: 10.1093/ije/dyh063
29. Parks MJ, Dodoo FN-A, Ayernor PK. Applying neighborhood-effect research to a global south city: A case study of collective efficacy in Accra, Ghana's low-income areas. *The Global South* 2014;8(2):119-38.
30. Pearce N. The ecological fallacy strikes back. *J Epidemiol Community Health* 2000;54(5):326. doi: 10.1136/jech.54.5.326
31. Burstein R, Henry NJ, Collison ML, et al. Mapping 123 million neonatal, infant and child deaths between 2000 and 2017. *Nature* 2019;574(7778):353-58. doi: 10.1038/s41586-019-1545-0
32. Arku RE, Bennett JE, Castro MC, et al. Geographical inequalities and social and environmental risk factors for under-five mortality in Ghana in 2000 and 2010: Bayesian spatial analysis of census data. *PLoS Med* 2016;13(6):e1002038. doi: 10.1371/journal.pmed.1002038
33. United Nations. Transforming Our World: The 2030 agenda for sustainable development. New York: UN, 2015.
34. Bhutta ZA. Mapping the geography of child mortality: A key step in addressing disparities. *Lancet Glob Health* 2016;4(12):e877-e78. doi: 10.1016/S2214-109X(16)30264-9
35. Ghana Statistical Service (GSS). Ghana Population and Housing Census. In: Service GS, ed. Accra, Ghana, 2010.
36. Owusa G. Decentralized development planning and fragmentation of metropolitan regions: the case of the Greater Accra Metropolitan Area, Ghana. *Ghana Journal of Geography* 2015;7:1-24.

For peer review only - http://bmjopen.bmj.com/site/about/guidelines.xhtml

37. Yankson P, Bertrand M. Challenges of urbanization in Ghana. In: Ardayfio-Schandorf E, ed. The mobile city of Accra: urban families, housing and residential places 2012:25-46.
38. Akubia J, Bruns A. Unravelling the frontiers of urban growth: Spatio-temporal dynamics of land-use change and urban expansion in Greater Accra Metropolitan Area, Ghana. *Land* 2019;8(9):131.
39. Kayode GA, Grobbee DE, Koduah A, et al. Temporal trends in childhood mortality in Ghana: Impacts and challenges of health policies and programs. *Glob Health Action* 2016;9:31907-07. doi: 10.3402/gha.v9.31907
40. Ghana National Health Insurance Act (Act 650), 2003.
41. Blanchet NJ, Fink G, Osei-Akoto I. The effect of Ghana's National Health Insurance Scheme on health care utilisation. *Ghana Med J* 2012;46(2):76-84.
42. Ministry of Health Ghana. Guidelines for implementing the exemption policy on maternal deliveries. Accra, Ghana: Ministry of Health, Ghana, 2004.
43. Ministry of Health Ghana. Under five's child health policy: 2007-2015. Accra, Ghana: Ministry of Health, Ghana, 2007.
44. Witter S, Garshong B, Ridde V. An exploratory study of the policy process and early implementation of the free NHIS coverage for pregnant women in Ghana. *Int J Equity Health* 2013;12(1):16. doi: 10.1186/1475-9276-12-16
45. Ghana Statistical Service (GSS), Ghana Health Service (GHS), ICF International. Ghana Demographic and Health Survey 2014. Rockville, Maryland, USA: GSS, GHS, and ICF International, 2015.
46. Rajaratnam JK, Tran LN, Lopez AD, et al. Measuring under-five mortality: validation of new low-cost methods. *PLoS Med* 2010;7(4):e1000253. doi: 10.1371/journal.pmed.1000253
47. Verhulst A. Child mortality estimation: An assessment of summary birth history methods using microsimulation. *Demogr Res* 2016;34:1075-128. doi: 10.4054/DemRes.2016.34.39
48. Hill K, You D, Inoue M, et al. Child mortality estimation: Accelerated progress in reducing global child mortality, 1990–2010. *PLoS Med* 2012;9(8):e1001303. doi: 10.1371/journal.pmed.1001303
49. Besag J, York J, Mollié A. Bayesian image restoration, with two applications in spatial statistics. *Ann Inst Stat Math* 1991;1:1-20. doi: 10.1007/BF00116466
50. Deaton A. The analysis of household surveys: a microeconometric approach to development policy. Baltimore, MD: Published for the World Bank [by] Johns Hopkins University Press, 1997.
51. Deaton A, Zaidi S. Guidelines for constructing consumption aggregates for welfare analysis. LSMS Working Paper No. 135. Washington, D.C.: World Bank 2002.
52. Cavanaugh A, Bixby H, Schmidt AM, et al. Locating poverty and inequality: an application of small area estimation methods using survey and census data from Ghana. *Manuscript in preparation* 2021
53. Owusu G. Social effects of poor sanitation and waste management on poor urban communities: A neighborhood-specific study of Sabon Zongo, Accra. *J Urban* 2010;3(2):145-60. doi: 10.1080/17549175.2010.502001
54. Guiteras R, Levinsohn J, Mobarak AM. Encouraging sanitation investment in the developing world: A cluster-randomized trial. *Science* 2015;348(6237):903-06. doi: 10.1126/science.aaa0491
55. Zhou Z, Dionisio KL, Verissimo TG, et al. Chemical composition and sources of particle pollution in affluent and poor neighborhoods of Accra, Ghana. *Environ Res Lett* 2013;8(4):044025. doi: 10.1088/1748-9326/8/4/044025

For peer review only

56. Jorgenson AK, Rice J. Urban slum growth and human health: a panel study of infant and child mortality in less-developed countries, 1990–2005. *J Poverty* 2010;14(4):382-402. doi: 10.1080/10875549.2010.517073
57. Rice J, Rice JS. The concentration of disadvantage and the rise of an urban penalty: urban slum prevalence and the social production of health inequalities in the developing countries. *Int J Health Serv* 2009;39(4):749-70. doi: 10.2190/HS.39.4.i [published Online First: 2009/11/26]
58. Ezeh A, Oyebode O, Satterthwaite D, et al. The history, geography, and sociology of slums and the health problems of people who live in slums. *Lancet* 2017;389(10068):547-58. doi: 10.1016/S0140-6736(16)31650-6
59. Kimani-Murage EW, Fotso JC, Egondi T, et al. Trends in childhood mortality in Kenya: The urban advantage has seemingly been wiped out. *Health Place* 2014;29:95-103. doi: https://doi.org/10.1016/j.healthplace.2014.06.003
60. Jankowska MM, Weeks JR, Engstrom R. Do the most vulnerable people live in the worst slums? A spatial analysis of Accra, Ghana. *Ann GIS* 2011;17(4):221-35. doi: 10.1080/19475683.2011.625976
61. Jankowska MM, Benza M, Weeks JR. Estimating spatial inequalities of urban child mortality. *Demogr Res* 2013;28:33-62. doi: 10.4054/DemRes.2013.28.2
62. Hewett PC, Montgomery MR. Poverty and public services in developing-country cities. Policy Research Division Working Paper No. 154. New York: Population Council 2001.
63. Temin M, Montgomery M, Engebretsen S, et al. Girls on the move: adolescent girls & migration in the developing world. New York: Population Council, 2013.
64. UNICEF, Institute of Statistical Social and Economic Research (ISSER) University of Ghana, Carolina Population Center University of North Carolina at Chapel Hill, et al. Ghana LEAP 1000 Programme: Endline Evaluation Report. Ghana: Ministry of Gender, Children and Social Protection, 2018.
65. Toure S, Weeks J, Lopez-Carr D, et al. Evaluating links between dynamic urban landscapes and under-five child mortality in Accra, Ghana. *Demogr Res* 2020;42(20):589-614. doi: 10.4054/DemRes.2020.42.20
66. Grant R, Yankson P. Accra. *Cities* 2003;20(1):65-74. doi: 10.1016/s0264-2751(02)00090-2
67. Hill K, Lopez AD, Shibuya K, et al. Interim measures for meeting needs for health sector data: births, deaths, and causes of death. *Lancet* 2007;370(9600):1726-35. doi: 10.1016/S0140-6736(07)61309-9
68. Silva R. Child mortality estimation: Consistency of under-five mortality rate estimates using full birth histories and summary birth histories. *PLoS Med* 2012;9(8):e1001296. doi: 10.1371/journal.pmed.1001296
69. Li Z, Hsiao Y, Godwin J, et al. Changes in the spatial distribution of the under-five mortality rate: small-area analysis of 122 DHS surveys in 262 subregions of 35 countries in Africa. *PLoS One* 2019;14(1):e0210645. doi: 10.1371/journal.pone.0210645
70. Bhutta ZA, Das JK, Bahl R, et al. Can available interventions end preventable deaths in mothers, newborn babies, and stillbirths, and at what cost? *Lancet* 2014;384(9940):347-70. doi: 10.1016/s0140-6736(14)60792-3
71. Keats EC, Das JK, Salam RA, et al. Effective interventions to address maternal and child malnutrition: An update of the evidence. *Lancet Child Adolesc Health* 2021 doi: 10.1016/s2352-4642(20)30274-1
72. Eilerts H, Romero Prieto J, Eaton JW, et al. Age patterns of under-5 mortality in sub-Saharan Africa during 1990–2018: A comparison of estimates from demographic

For peer review only - http://bmjopen.bmj.com/site/about/guidelines.xhtml

surveillance with full birth histories and the historic record. *Demogr Res* 2021;44:415-42. doi: 10.4054/DemRes.2021.44.18

73. Lopez AD, McLaughlin D, Richards N. Reducing ignorance about who dies of what: Research and innovation to strengthen CRVS systems. *BMC Med* 2020;18(1):58. doi: 10.1186/s12916-020-01526-9 [published Online First: 2020/03/10]
74. Kwan M-P. The limits of the neighborhood effect: Contextual uncertainties in geographic, environmental health, and social science research. *Ann Am Assoc Geogr* 2018;108(6):1482-90. doi: 10.1080/24694452.2018.1453777
75. United Nations. The Millennium Development Goals report 2015. New York: UN, 2015.
76. Burke M, Heft-Neal S, Bendavid E. Sources of variation in under-5 mortality across sub-Saharan Africa: A spatial analysis. *Lancet Glob Health* 2016;4(12):e936-e45. doi: 10.1016/s2214-109x(16)30212-1 [published Online First: 2016/10/30]
77. Earth Institute Millennium Cities Initiative. AMA community upgrading profile: Nima-Maamobi drain area. New York: The Earth Institute at Columbia University, 2012.
78. Roder-DeWan S, Nimako K, Twum-Danso NAY, et al. Health system redesign for maternal and newborn survival: rethinking care models to close the global equity gap. *BMJ Glob Health* 2020;5(10):e002539. doi: 10.1136/bmjgh-2020-002539 [published Online First: 2020/10/16]
79. Kruk ME, Gage AD, Joseph NT, et al. Mortality due to low-quality health systems in the universal health coverage era: A systematic analysis of amenable deaths in 137 countries. *Lancet* 2018;392(10160):2203-12. doi: 10.1016/s0140-6736(18)31668-4
80. Bhutta ZA, Das JK, Walker N, et al. Interventions to address deaths from childhood pneumonia and diarrhoea equitably: what works and at what cost? *Lancet* 2013;381(9875):1417-29. doi: 10.1016/S0140-6736(13)60648-0
81. Campbell OMR, Calvert C, Testa A, et al. The scale, scope, coverage, and capability of childbirth care. *Lancet* 2016;388(10056):2193-208. doi: 10.1016/s0140-6736(16)31528-8

For peer review only

Table 1 Population and household characteristics of neighbourhoods in the Greater Accra Metropolitan Area and correlations with neighbourhood under-five mortality in 2010. Median levels across all, peri-urban and urban neighbourhoods presented with interquartile range in parentheses. Spearman's rank correlation coefficients show the correlation between neighbourhood under-five mortality and neighbourhood characteristics that indicate the socio-economic and living conditions. Supplementary appendix 4 shows the distribution of under-five mortality across quintiles of analysed indicators.

	Median (inter-quartile range)			Correlation coefficient (Spearman's rho)		
Neighbourhood population and household characteristics	all	peri-urban	urban	all	peri-urban	urban
Child mortality rate (deaths per 1,000 live births)	80 (68-92)	69 (57-79)	90 (79-99)	n/a	n/a	n/a
Population	2972 (767-12798)	730 (439-1380)	11754 (6254-20873)	n/a	n/a	n/a
Population aged under five years	384 (107-1430)	105 (64-195)	1267 (703-2486)	n/a	n/a	n/a
Median household consumption (GHS)	5599 (4249-6894)	4485 (3616-5432)	6690 (5633-7473)	0.44*	-0.18*	0.35*
Population density (per km^2)	1858 (614-6366)	682 (156-1405)	5610 (1914-14513)	0.44*	0.04	0.09
Women's s literacy[a]	90.2% (83.2%-93.3%)	88.0% (68.9%-93.0%)	91.4% (88.2%-93.4%)	0.03	-0.41*	0.12
Women's schooling: at least primary[a]	90.2% (83.2%-93.3%)	88.0% (68.9%-93.0%)	91.4% (88.2%-93.4%)	0.03	-0.41*	0.12

For peer review only - http://bmjopen.bmj.com/site/about/guidelines.xhtml

For peer review only

Neighbourhood population and household characteristics	Median (inter-quartile range) all	peri-urban	urban	Correlation coefficient (Spearman's rho) all	peri-urban	urban
Women's schooling: at least middle[a]	78.7% (67.9%-83.4%)	74.9% (43.3%-81.9%)	80.4% (76.0%-84.2%)	0.09	-0.41*	0.14*
Women's schooling: at least secondary[a]	36.6% (24.5%-44.3%)	30.7% (6.4%-39.8%)	40.6% (33.3%-47.7%)	0.18*	-0.38*	0.21*
Women's schooling: post-secondary[a]	10.5% (4.9%-15.4%)	7.4% (1.2%-12.5%)	12.6% (8.3%-17.6%)	0.18*	-0.30*	0.17*
Employment[b]	67.4% (64.7%-70.5%)	68.2% (64.5%-72.9%)	67.1% (64.9%-68.9%)	-0.01	0.31*	-0.18*
Occupation: primary sector[b]	2.0% (1.5%-3.8%)	3.4% (1.6%-21.4%)	1.8% (1.4%-2.5%)	-0.04	0.44*	-0.03
Occupation: non-primary sector[b]	64.0% (60.3%-66.6%)	61.7% (37.5%-65.9%)	64.9% (62.6%-66.7%)	0.04	-0.25*	-0.14*
Non-agricultural household[c]	94.4% (88.8%-96.4%)	89.1% (47.2%-95.3%)	95.3% (93.3%-96.9%)	0.10*	-0.40*	0.03
Improved dwelling type[c]	88.1% (79.4%-93.4%)	88.5% (78.1%-94.7%)	88.1% (80.0%-92.3%)	-0.13*	-0.28*	0.02
Improved drinking water source[c]	95.1% (85.4%-97.9%)	92.8% (78.6%-99.0%)	95.9% (89.7%-97.6%)	-0.11*	-0.39*	-0.02
Improved water source[c]	89.5% (71.8%-96.7%)	80.5% (50.6%-94.6%)	93.7% (81.7%-97.1%)	0.12*	-0.18*	0.07
Improved toilet facilities[c]	70.2% (48.3%-84.6%)	74.2% (51.2%-89.7%)	66.5% (47.3%-80.6%)	-0.18*	-0.29*	0.00
Improved solid waste disposal[c]	77.4% (54.2%-90.8%)	64.4% (36.6%-86.3%)	85.7% (68.6%-93.4%)	0.34*	0.05	0.26*

For peer review only - http://bmjopen.bmj.com/site/about/guidelines.xhtml

Neighbourhood population and household characteristics	Median (inter-quartile range) all	peri-urban	urban	Correlation coefficient (Spearman's rho) all	peri-urban	urban
Improved liquid waste disposal[c]	26.3% (10.2%-40.0%)	12.7% (1.9%-26.5%)	32.9% (23.8%-48.3%)	0.31*	-0.25*	0.27*
Improved cooking fuel use[c]	45.0% (27.4%-55.2%)	35.9% (4.8%-50.2%)	49.2% (39.4%-57.9%)	0.12*	-0.37*	0.13*
Improved lighting source[c]	83.9% (71.9%-92.0%)	79.8% (55.2%-87.6%)	88.4% (77.6%-93.3%)	0.05	-0.48*	0.1
Improved floor material[c]	90.9% (84.6%-94.1%)	92.1% (84.4%-96.2%)	90.3% (84.9%-93.0%)	-0.14*	-0.18*	-0.05
Improved roof material[c]	95.6% (91.2%-97.2%)	95.2% (79.8%-98.3%)	95.6% (93.6%-96.7%)	-0.25*	-0.43*	-0.23*
Improved wall material[c]	87.2% (76.0%-92.4%)	85.9% (59.2%-93.8%)	87.6% (83.0%-91.7%)	-0.22*	-0.50*	-0.18*

* significant at the 5% level.

[a] measured as the proportion of women of childbearing age (15-49 years) in the neighbourhood.

[b] measured as the proportion of population of working age (15-64 years) in the neighbourhood.

[c] measured as the proportion of population in the neighbourhood.

For peer review only - http://bmjopen.bmj.com/site/about/guidelines.xhtml

Figure 1. The Greater Accra Metropolitan Area with neighbourhood boundaries shown in grey and district boundaries shown in black (grey) (source: Ghana Statistical service). Urban neighbourhoods are shown in purple and peri-urban neighbourhoods shown in green. The inset shows the location of GAMA in Ghana and Western Africa.

For peer review only

For peer review only - http://bmjopen.bmj.com/site/about/guidelines.xhtml

Figure 2. a) Neighbourhood under-five mortality in the Greater Accra Metropolitan Area in 2010, by district. The black vertical line and box show the median and interquartile range of under-five mortality across neighbourhoods in a district and the red vertical line shows the mean. b) The contribution of neighbourhoods in each district to each decile of neighbourhood under-five mortality. Decile 1 groups the 10% of neighbourhoods with the lowest under-five mortality rates and decile 10 groups the 10% of neighbourhoods with the highest under-five mortality rates.

For peer review only

Figure 3. Under-five mortality rates in neighbourhoods of the Greater Accra Metropolitan Area in 2010. Neighbourhood boundaries are shown in grey, district boundaries are shown in black. The colour scale diverges at the mean under-five mortality rate across all GAMA neighbourhoods. Neighbourhoods with U5M above the GAMA average are shown in orange; neighbourhoods with U5M below the GAMA average are shown in purple.

For peer review only

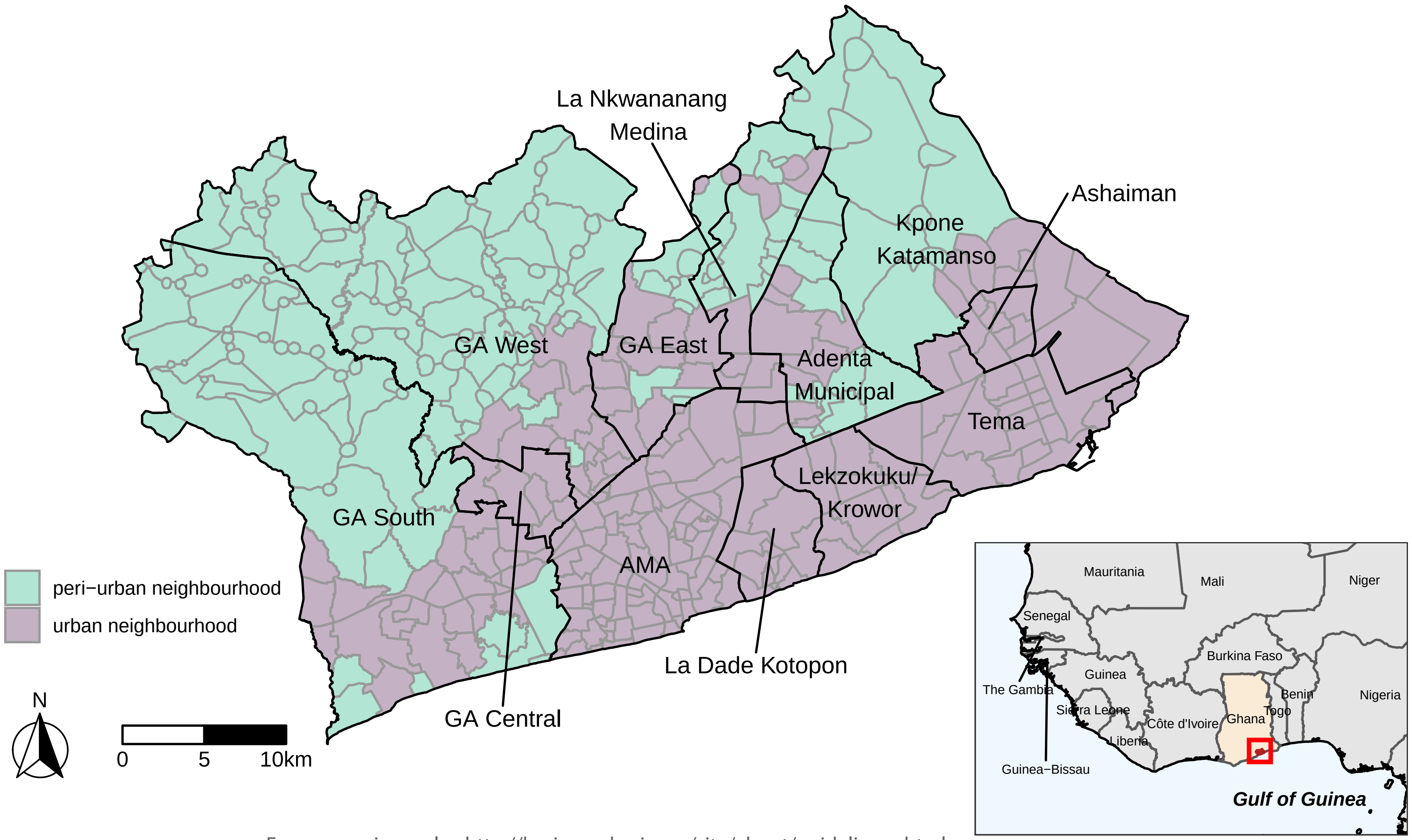
La Nkwananang
Medina
Ashaiman
Kpone
Katamanso
GA West
GA East
Adenta
Municipal
Tema
Lekzokuku/
Krowor
GA South
AMA
La Dade Kotopon
GA Central
peri−urban neighbourhood
urban neighbourhood
N
0
5
10km
Mauritania
Mali
Niger
Senegal
Burkina Faso
Guinea
The Gambia
Benin
Nigeria
Sierra Leone
Togo
Côte d'Ivoire
Ghana
Liberia
Guinea−Bissau
Gulf of Guinea

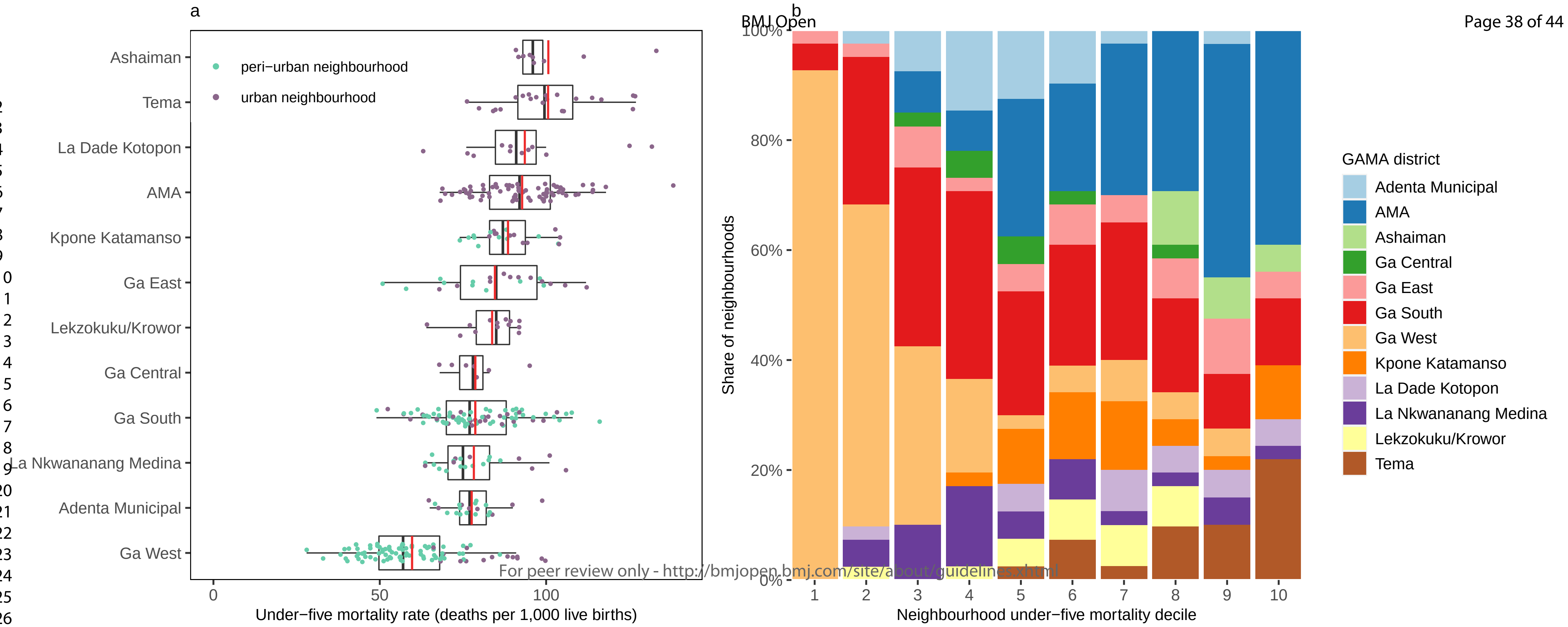

For peer review only - http://bmjopen.bmj.com/site/about/guidelines.xhtml

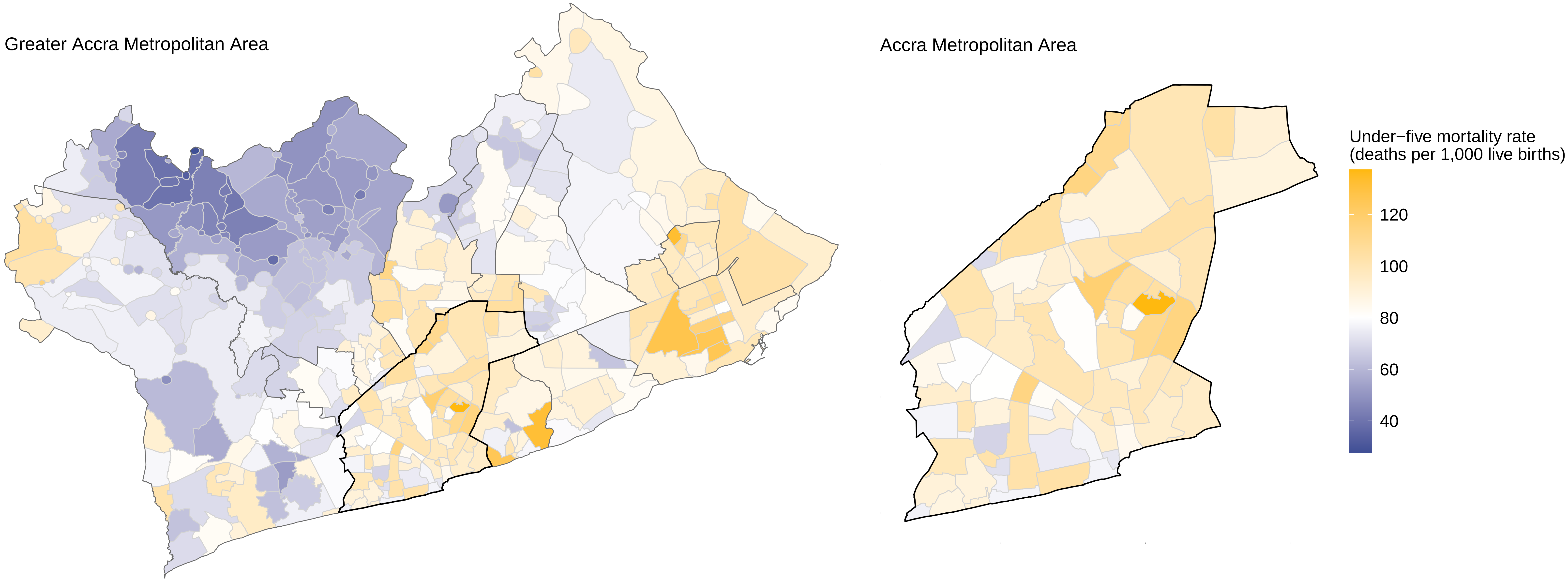
Greater Accra Metropolitan Area
Accra Metropolitan Area
Under−five mortality rate
(deaths per 1,000 live births)
120
100
80
60
40

Supplementary appendix 1 Relationship between number of children reported dead by women in a neighbourhood (as a proportion of live births) and the average number live births per woman, by age group of women. Each point represents a neighbourhood, coloured by district.

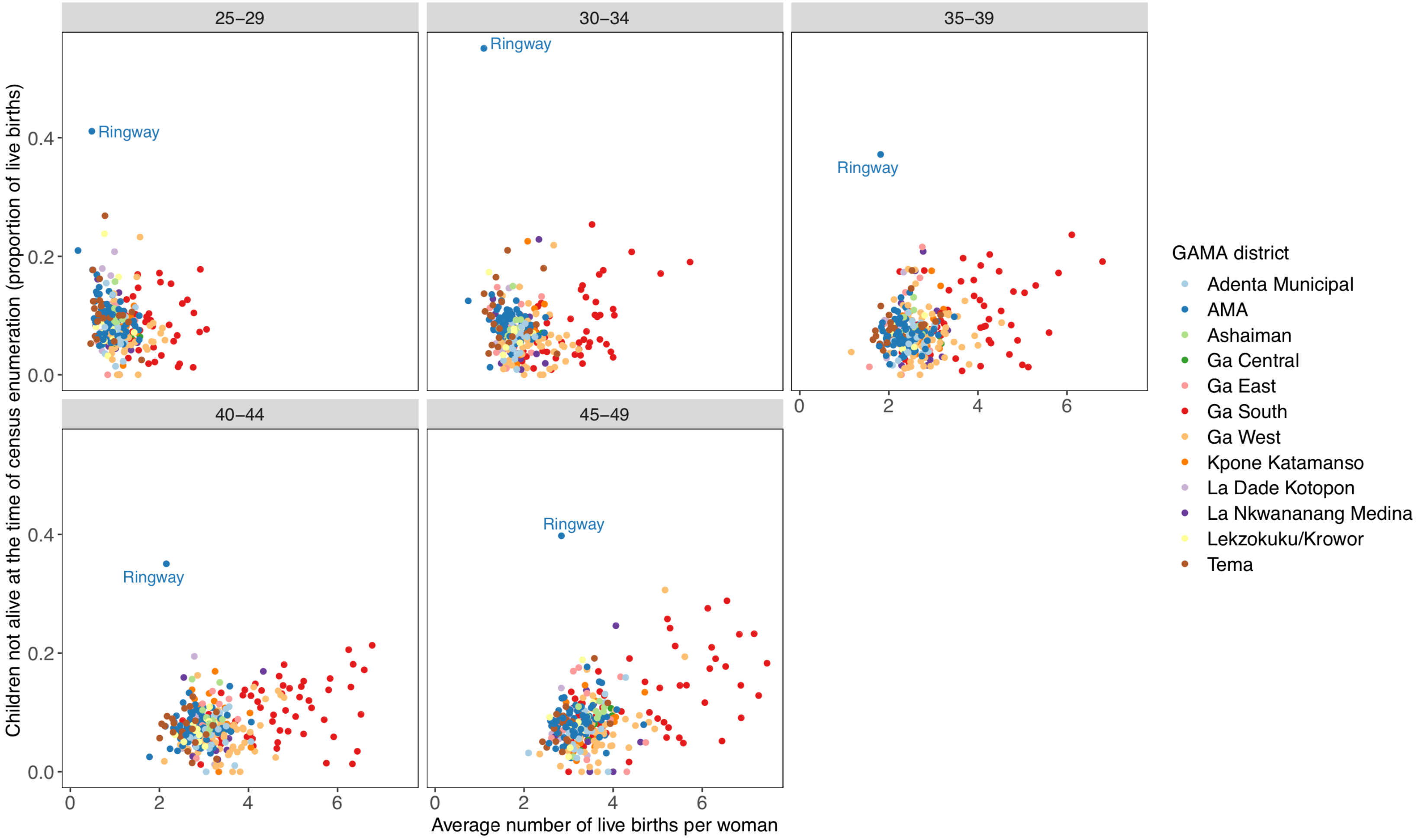

For each age group of women, only neighbourhoods in which women collectively reported more than 50 live births are shown. The Ringway neighbourhood was a consistent outlier across age groups of women and therefore we did not include the summary birth history data of women living in Ringway in our analysis. Reported estimates for Ringway were therefore informed only by data from surrounding neighbourhoods.

Supplementary appendix 2. 'Improved' or 'unimproved classification of census responses to questions on household characteristics.

Household characteristic	Census response 'improved'	Census response 'unimproved'
Dwelling type	Compound house (rooms)<br>Flat/Apartment<br>Semi-detached house<br>Separate house	Huts/Buildings (different compound)<br>Huts/Buildings (same compound)<br>Improvised home (kiosk/container, etc.)<br>Living quarters attached to office/shop<br>Tent<br>Uncompleted building<br>Other
Drinking water source	Bore-hole/Pump/Tube well<br>Bottled water<br>Pipe-borne inside dwelling<br>Pipe-borne outside dwelling<br>Protected spring<br>Protected well<br>Public tap/Standpipe<br>Sachet water	Dugout/Pond/Lake/Dam/Canal<br>Rainwater<br>River/Stream<br>Tanker supply/Vendor provided<br>Unprotected spring<br>Unprotected well<br>Other
Water source	Bore-hole/Pump/Tube well<br>Pipe-borne inside dwelling<br>Pipe-borne outside dwelling<br>Protected spring<br>Protected well<br>Public tap/Standpipe	Dugout/Pond/Lake/Dam/Canal<br>Rainwater<br>River/Stream<br>Tanker supply/Vendor provided<br>Unprotected spring<br>Unprotected well<br>Other
Toilet facilities	KVIP<br>Pit latrine<br>W.C.	Bucket/Pan<br>Public toilet (WC, KVIP, Pit, Pan, etc.)<br>No facilities (bush/beach/field)<br>Other
Solid waste disposal	Collected<br>Public dump (container)<br>Public dump (open space)	Buried by household<br>Burned by household<br>Dumped indiscriminately<br>Other
Liquid waste disposal	Through drainage into a pit (soak away)<br>Through drainage system into a gutter<br>Through the sewerage system	Thrown into gutter<br>Thrown onto compound<br>Thrown onto the street/outside<br>Other
Cooking fuel use	Electricity<br>Gas<br>Kerosene	Animal waste<br>Charcoal<br>Crop residue<br>Saw dust<br>Wood<br>None, no cooking<br>Other
Lighting source	Electricity (mains)<br>Electricity (private generator)<br>Solar energy	Candle<br>Crop residue<br>Firewood<br>Flashlight/Torch<br>Gas lamp<br>Kerosene lamp<br>Other
Floor material	Burnt brick<br>Cement/Concrete<br>Ceramic/Porcelain/Granite/Marble tiles<br>Stone<br>Terrazzo/Terrazzo tiles<br>Vinyl tiles	Earth/Mud<br>Wood<br>Other
Roof material	Cement/Concrete<br>Metal sheet<br>Roofing tile<br>Slate/Asbestos	Bamboo<br>Mud/Mud bricks/Earth<br>Thatch/Palm leaf or Raffia<br>Wood<br>Other
Wall material	Burnt bricks<br>Cement blocks/Concrete<br>Landcrete<br>Metal sheet/Slate/Asbestos<br>Stone	Bamboo<br>Mud brick/Earth<br>Palm leaf/Thatch (grass)/Raffia<br>Wood<br>Other

For peer review only

Supplementary appendix 3. Under-five mortality rates in neighbourhoods of the Greater Accra Metropolitan Area from sensitivity analyses replacing zero MAC-5q0 estimates with **a)** 0.0001 and **b)** the minimum estimated non-zero 5q0 (0.00632). Neighbourhood boundaries are shown in grey, district boundaries are shown in black. The colour scale diverges at the mean under-five mortality rate across all GAMA neighbourhoods. Neighbourhoods with U5M above the GAMA average are shown in orange; neighbourhoods with U5M below the GAMA average are shown in purple.

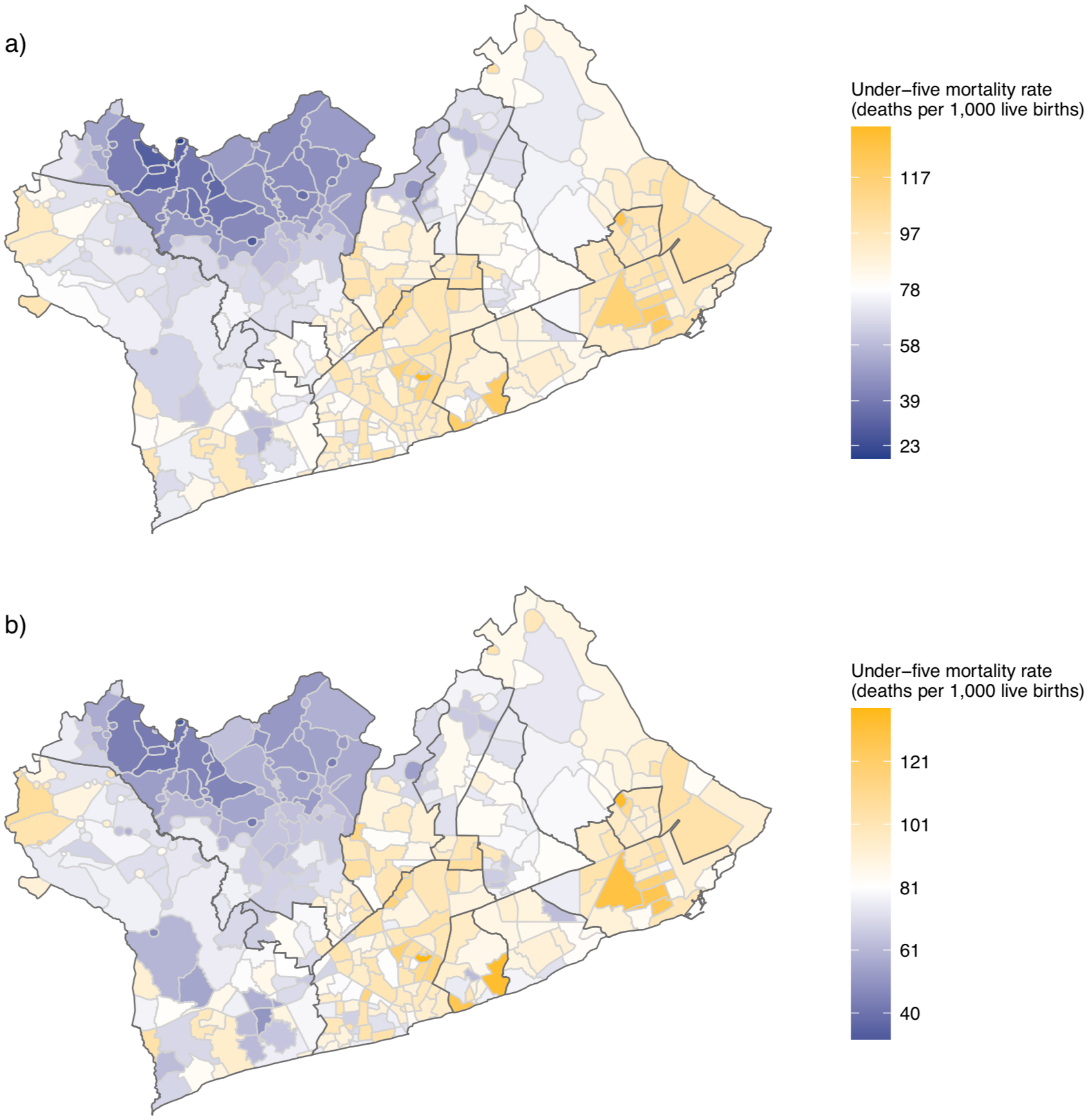

Supplementary appendix 4: The distribution of under−five mortality at increasing levels of neighbourhood living and socio−economic condition indicators.

Neighbourhoods are grouped into quintiles based on the measured indicator, separately by urban or peri−urban area.
The horizontal line and box show the median and interquartile range.

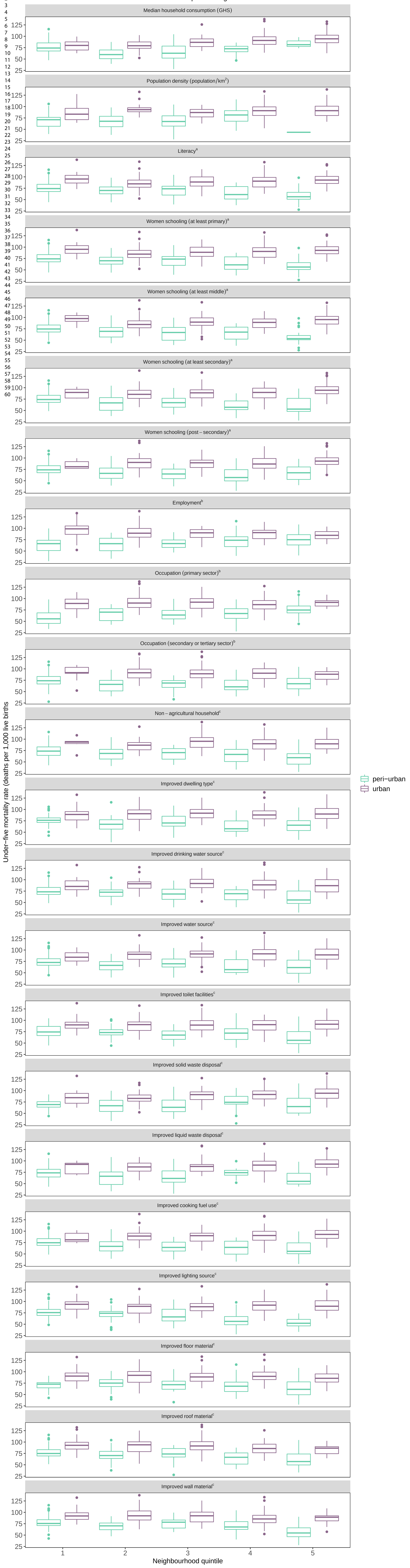

Neighbourhood indicator measured as
(a) the proportion of women of childbearing age;
(b) the proportion of population of working age (15–64 years); and,
(c) the proportion of total population.

STROBE Statement—checklist of items that should be included in reports of observational studies

	Item No	Recommendation	Reported on Page No
**Title and abstract**	1	(*a*) Indicate the study's design with a commonly used term in the title or the abstract	1,2,3
		(*b*) Provide in the abstract an informative and balanced summary of what was done and what was found	2,3
**Introduction**			
Background/rationale	2	Explain the scientific background and rationale for the investigation being reported	5,6
Objectives	3	State specific objectives, including any prespecified hypotheses	6,7
**Methods**			
Study design	4	Present key elements of study design early in the paper	6
Setting	5	Describe the setting, locations, and relevant dates, including periods of recruitment, exposure, follow-up, and data collection	7,8
Participants	6	(*a*) *Cohort study*—Give the eligibility criteria, and the sources and methods of selection of participants. Describe methods of follow-up<br>*Case-control study*—Give the eligibility criteria, and the sources and methods of case ascertainment and control selection. Give the rationale for the choice of cases and controls<br>*Cross-sectional study*—Give the eligibility criteria, and the sources and methods of selection of participants	n/a
		(*b*) *Cohort study*—For matched studies, give matching criteria and number of exposed and unexposed<br>*Case-control study*—For matched studies, give matching criteria and the number of controls per case	n/a
Variables	7	Clearly define all outcomes, exposures, predictors, potential confounders, and effect modifiers. Give diagnostic criteria, if applicable	9,10
Data sources/ measurement	8*	For each variable of interest, give sources of data and details of methods of assessment (measurement). Describe comparability of assessment methods if there is more than one group	9,10
Bias	9	Describe any efforts to address potential sources of bias	10
Study size	10	Explain how the study size was arrived at	7,9
Quantitative variables	11	Explain how quantitative variables were handled in the analyses. If applicable, describe which groupings were chosen and why	7,9,10,12
Statistical methods	12	(*a*) Describe all statistical methods, including those used to control for confounding	9,10,11,12,13
		(*b*) Describe any methods used to examine subgroups and interactions	
		(*c*) Explain how missing data were addressed	n/a
		(*d*) *Cohort study*—If applicable, explain how loss to follow-up was addressed<br>*Case-control study*—If applicable, explain how matching of cases and controls was addressed<br>*Cross-sectional study*—If applicable, describe analytical methods taking account of sampling strategy	n/a
		(*e*) Describe any sensitivity analyses	11

For peer review only - http://bmjopen.bmj.com/site/about/guidelines.xhtml

Results			Reported on Page No
Participants	13*	(a) Report numbers of individuals at each stage of study—eg numbers potentially eligible, examined for eligibility, confirmed eligible, included in the study, completing follow-up, and analysed	13
		(b) Give reasons for non-participation at each stage	n/a
		(c) Consider use of a flow diagram	n/a
Descriptive data	14*	(a) Give characteristics of study participants (eg demographic, clinical, social) and information on exposures and potential confounders	30,31,32
		(b) Indicate number of participants with missing data for each variable of interest	n/a
		(c) *Cohort study*—Summarise follow-up time (eg, average and total amount)	n/a
Outcome data	15*	*Cohort study*—Report numbers of outcome events or summary measures over time	n/a
		Case-control study—Report numbers in each exposure category, or summary measures of exposure	n/a
		Cross-sectional study—Report numbers of outcome events or summary measures	13,14
Main results	16	(*a*) Give unadjusted estimates and, if applicable, confounder-adjusted estimates and their precision (eg, 95% confidence interval). Make clear which confounders were adjusted for and why they were included	n/a
		(*b*) Report category boundaries when continuous variables were categorized	n/a
		(*c*) If relevant, consider translating estimates of relative risk into absolute risk for a meaningful time period	n/a
Other analyses	17	Report other analyses done—eg analyses of subgroups and interactions, and sensitivity analyses	14,15
**Discussion**			
Key results	18	Summarise key results with reference to study objectives	15,16
Limitations	19	Discuss limitations of the study, taking into account sources of potential bias or imprecision. Discuss both direction and magnitude of any potential bias	19,20
Interpretation	20	Give a cautious overall interpretation of results considering objectives, limitations, multiplicity of analyses, results from similar studies, and other relevant evidence	16,17,18,20,21,22
Generalisability	21	Discuss the generalisability (external validity) of the study results	21
**Other information**			
Funding	22	Give the source of funding and the role of the funders for the present study and, if applicable, for the original study on which the present article is based	23

*Give information separately for cases and controls in case-control studies and, if applicable, for exposed and unexposed groups in cohort and cross-sectional studies.

Note: An Explanation and Elaboration article discusses each checklist item and gives methodological background and published examples of transparent reporting. The STROBE checklist is best used in conjunction with this article (freely available on the Web sites of PLoS Medicine at http://www.plosmedicine.org/, Annals of Internal Medicine at http://www.annals.org/, and Epidemiology at http://www.epidem.com/). Information on the STROBE Initiative is available at www.strobe-statement.org.

For peer review only - http://bmjopen.bmj.com/site/about/guidelines.xhtml
